# Power and optimal study design in iPSC-based brain disease modelling

**DOI:** 10.1038/s41380-022-01866-3

**Published:** 2022-11-16

**Authors:** Jessie W. Brunner, Hanna C. A. Lammertse, Annemiek A. van Berkel, Frank Koopmans, Ka Wan Li, August B. Smit, Ruud F. Toonen, Matthijs Verhage, Sophie van der Sluis

**Affiliations:** 1 Dept. Functional Genomics, CNCR, VU University Amsterdam, 1081 HV Amsterdam, The Netherlands; 2 Functional Genomics, Department of Human Genetics, CNCR, Amsterdam, UMC 1081 HV Amsterdam, The Netherlands; 3 Dept. Molecular & Cellular Neurobiology, CNCR, VU University Amsterdam, 1081 HV Amsterdam, The Netherlands; 4 Dept. Complex Trait Genetics, CNCR, VU University Amsterdam, 1081 HV Amsterdam, The Netherlands; 5 Dept. of Child and Adolescence Psychiatry, section Complex Trait Genetics, Amsterdam UMC, Amsterdam, The Netherlands

**Keywords:** Neuroscience, Diseases, Stem cells, Biological techniques

## Abstract

Studies using induced pluripotent stem cells (iPSCs) are gaining momentum in brain disorder modelling, but optimal study designs are poorly defined. Here, we compare commonly used designs and statistical analysis for different research aims. Furthermore, we generated immunocytochemical, electrophysiological, and proteomic data from iPSC-derived neurons of five healthy subjects, analysed data variation and conducted power simulations. These analyses show that published case–control iPSC studies are generally underpowered. Designs using isogenic iPSC lines typically have higher power than case–control designs, but generalization of conclusions is limited. We show that, for the realistic settings used in this study, a multiple isogenic pair design increases absolute power up to 60% or requires up to 5-fold fewer lines. A free web tool is presented to explore the power of different study designs, using any (pilot) data.

## Introduction

Induced pluripotent stem cells (iPSCs) greatly facilitate the investigation of human disease mechanisms, the characterization of patient-specific phenotypes, and the development of new, personalized treatments. For brain disorders, iPSC-based disease modelling is particularly advantageous given the highly restricted access to primary tissue. However, considerations regarding choice and optimization of study designs remain underexposed and consequently the impact of iPSC-based disease modelling on scientific progress has probably not reached its full potential yet.

Various study designs are being applied in iPSC-based disease modelling: classical case–control studies comparing unrelated patients to controls, and gene-editing studies comparing known genetic variants against isogenic controls, either by introducing such variants in standard control lines or by repairing the disease mutation in patient-derived iPSCs. These designs differ in the research questions they address, generalizability to patient populations, and applicability for polygenic/idiopathic cases. In addition, they require different statistical approaches and different sample sizes to obtain adequate statistical power. Suboptimal study design choices, underpowered studies, and/or incorrect statistical analyses all reduce the chance of detecting true effects, and bias the estimates of true effects. In this way, the reliability of study results is diminished and the potential impact of iPSC-based studies is limited. Unfortunately, “power failure” is a general and ubiquitous problem in neuroscience: meta-analyses indicate that the majority of studies across neuroscience subfields has inadequate statistical power [1, 2]. Additionally, statistical analyses in many neuroscience studies violate essential assumptions [3–8]. Hence, optimizing study designs and associated statistical analyses are crucial aspects for the optimal utilization of iPSC-based disease modelling in neuroscience.

Furthermore, several features of typical iPSC-based studies dictate specific adjustments of study designs and statistical analyses that are less common in traditional disease modelling. First, genetic heterogeneity between iPSC donors is much larger than for disease modelling using inbred animal models or single standard cell lines (for review, see [9]). Consequently, iPSC study designs require larger cohort sizes, which massively increases experimental burden (resources, time). In practice, however, many iPSC-based brain disease modelling studies published over the last five years are based on a very limited cohort sizes, as we will demonstrate below. Optimizing study designs minimizes these costs, while maintaining acceptable false positive/negative rates. Second, in most iPSC-based studies, multiple inductions (culture batches) are generated from each iPSC line and each batch contains multiple neurons, resulting in multiple observations (i.e., individual measurements or recordings) from the same iPSC line (i.e., individual). Such study designs generate clustered data, where different data points are not truly independent. If not statistically accounted for, such clustered data lead to severely inflated false positive rates [5, 6, 8]. This is particularly damaging for disease modelling, as false positive outcomes of preclinical studies are doomed to fail translation to the clinic [10], a conclusion that will often only be reached after expensive, risky, yet futile clinical trials. To take all these considerations into account and select optimal study designs for specific research questions, a systematic comparison of designs, and their attainable power, is required.

In this study, we provide a framework to conceive optimal, statistically rigorous iPSC-based studies. We discuss the applicability of different study designs, their data structure and appropriate statistical analysis, and present power estimations and sample size calculations for typical iPSC study designs based on real, representative data examples from iPSC-derived neurons of five healthy individuals. Additionally, we developed a web tool (https://jessiebrunner.shinyapps.io/App_PowerCurves/) to estimate statistical power for different iPSC study designs, sample sizes, and expected effect sizes. Together, these design considerations, the power calculations, and the web tool promote optimal iPSC study designs and statistically rigorous practices to maximize the future impact of iPSC technology.

## Materials and methods

### Laboratory animals

Glia were prepared from newborn P0-P1 pups from female Wister rats (Crl:WI, strain code 003). Animals were housed and bred according to institutional, Dutch and U.S. governmental guidelines.

### iPSC lines

Five iPSC lines from unrelated individuals with no diagnosed disease status were used for this study. An additional iPSC line of the same genetic background as line C2 was used, in which the NGN2 overexpression cassette was engineered into a safe harbour-locus. Details for each line are listed in the Supplementary Methods. iPSCs were routinely tested for mycoplasma contamination. Prior to induction of NGN2, iPSCs were subjected to SNP-array analysis and CNV calling as described in the Supplementary Methods.

### Generation of iPSC-derived neurons

Neuronal differentiation was induced as described previously [11] and details are provided in the Supplementary Methods. From all iPSC lines, neurons could reliably be induced, though line C5 produced a lower neuronal yield, so that insufficient numbers of neurons could be produced for electrophysiological and proteomic analyses. The induction process was repeated several times generating several culture batches from which data were acquired. After 39–45 days in vitro, at a time point where iPSC-derived neurons reliably show mature synaptic transmission [12], samples were obtained for proteomic profiling, coverslips with autaptic neurons were fixed for morphological analysis, or patch-clamp electrophysiological recordings were performed.

### Mass spectroscopy

Neurons were harvested in PBS with protease inhibitor, spinned down and resuspended in loading buffer. An SDS–PAGE LC-MS/MS approach was used for protein identification as described previously [13] and detailed in the Supplementary Methods.

A spectral library was made from pooled samples of all three lines, one for each culture condition, collected at two different time points (DIV15 and DIV42). Additionally, a pooled sample glia cultured without neurons, collected at DIV15 and DIV42, was included. Spectral library samples were measured in DDA mode and analyzed using MaxQuant 1.6.3.4 [14]. The Uniprot human reference proteome database (SwissProt + TrEMBL, version 2019-11) was used to annotate spectra. The minimum peptide length was set to 6, with at most two miss-cleavages allowed. Methionine oxidation and N-terminal acetylation were set as variable modifications with cysteine Propionamide set as fixed modification. For both peptide and protein identification a false discovery rate of 0.01 was set.

SWATH data were searched against the spectral library (peptides and proteins identified from DDA data by MaxQuant) using Spectronaut 13.7 [15] with default settings. The resulting abundance values and qualitative scores for each peptide in the spectral library were exported for further downstream analysis. Data were analysed as described in the Supplementary Methods.

### Immunocytochemistry and morphological analysis

Neurons were fixed with 3.7% paraformaldehyde, permeabilized with 0.5% Triton X-100 (Thermo Fisher #T/3751/08), and incubated in blocking buffer (PBS containing 2% normal goat serum (NGS; Thermo Fisher #11540526) and 0.1% Triton X-100) and stained with primary antibodies for 2 h at room temperature (RT). Primary antibodies used: chicken anti-MAP2 (1:500, Abcam Ab5392) and Synaptophysin 1 (1:1000, Synaptic Systems #101004). After three washes with PBS, neurons were stained with secondary antibodies Alexa Fluor (1:1000; Invitrogen) for 1 h at RT. Coverslips were washed three times with PBS and mounted on microscopic slides with Mowiol-DABCO. Images were acquired on a Nikon Ti-Eclipse microscope equipped with a confocal scanner model A1R+, using a 40X oil immersion objective (NA = 1.3; Carl Zeiss). Z stacks were acquired with 0.5 µM intervals. Confocal settings were kept constant between cultures. Z Stacks were collapsed to maximal projections for image analysis. Images were analysed in MATLAB with SynD [16]. Synapse detection settings were kept constant between cultures.

### Electrophysiology

Autaptic neurons were recorded in whole-cell voltage clamp mode between DIV42-47. For a detailed description of devices and solutions used, see Supplementary Methods. Resting membrane potential was measured in current-clamp immediately after break-in of the membrane. After this, neurons were maintained in voltage-clamp configuration at a holding potential of −70 mV. Spontaneous activity was recorded first, with a sampling frequency of 20 kHz (Bessel filter 5–6 kHz) to allow accurate quantification of the kinetics parameters of the spontaneous events. Subsequently, a series of stimulation protocols was recorded, as described in the Supplementary Methods. Data analysis and exclusion criteria are explained in the Supplementary Methods.

### Analysis of variation and comparison to published autapse datasets

Coefficient of variation (CoV) was calculated as standard deviation divided by the mean value, for each morphological and electrophysiological parameter of interest. For the proteomics data, the CoV was calculated for each protein and the median CoV per iPSC line was compared to published datasets. For comparison to the current dataset, values from several published mouse autapse datasets were used, as outlined in the Supplementary Methods. A Kruskal-Wallis ANOVA showed that there was no significant difference between the mean CoVs of the four control lines and the safe-harbor NGN2 line measured with electrophysiology in this study (*p* = 0.5194).

### Analysis of explained variation

The proportions of variance explained by culture batch were calculated by the R^2^_marginal_ [17]. The R^2^_marginal_ quantifies the proportion of variance explained by fixed factors in the model (in our model, culture batch) in a multi-level random effects model. For the proteomics data, proportions of explained variance were calculated at the protein-level for both culture conditions using the Bioconductor-package variancePartition in R [18] (Fig. 3C).

### Quantification and statistical analysis

Graphs were generated using GraphPad Prism (v. 8). Unless otherwise specified, boxplots show the median value, interquartile range, and whiskers including all values within 1.5 times IQR from the median (Tukey-style whiskers). Outliers, defined as values >3 standard deviations above or below the group mean, were excluded prior to analysis. For statistical analyses, data were standardized to meet the assumptions for linear mixed-effects models. Linear mixed-effects models were fitted to the standardized data using the *lme4* package in R (R version 3.6.3). Culture batch was included as a fixed factor in the model. The relative proportions of variance explained by the multilevel model were calculated using the MuMIn R package. The conditional intraclass correlation coefficient (conditional ICC, i.e., the ICC obtained from the model in which the batch effect is accounted for [17]; was calculated using the ‘icc’ function (*performance* package in R). ICC values deviating from 0 suggest that the variation in the total data set is at least partly due to the clusters having different means (as expressed by a non-zero intercept variance). The significance of the ICC was evaluated by testing the significance of the intercept variance using a chi-square test of which the p-value is divided by two (common when testing a variance term; [19]). For all tests, α was set to 0.05.

### Power analysis

Synapse density was selected as an example parameter for all power simulations. Synapse density data measured in this study were corrected for variation of culture batch, using a mean-centering method: each datapoint within a batch was subtracted from the mean of that batch and added with the overall mean of the dataset. This allowed simplifying the statistical models as described in Supplementary textbox 2 by removing the fixed factor “Batch” from the statistical model used in the power simulations. All power curves were plotted using the ggplot2 R-package [20]. The Supplementary Methods contain a detailed explanation of the approach and settings for the power analyses performed for each design.

## Results

### Differences between commonly used iPSC-based study designs

**Fig. 1:**
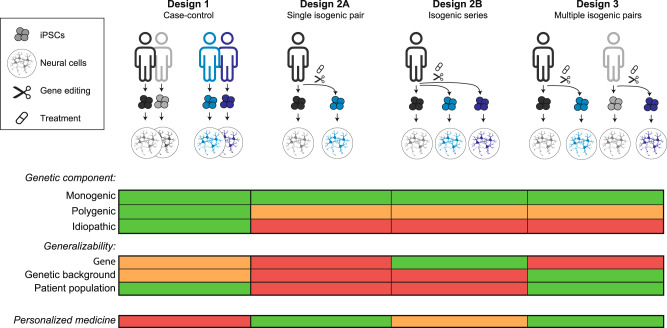
iPSC study designs address different research questions. Design 1 (case–control) is characterized by including iPSC lines from multiple independent donors per condition. For design 2A (single isogenic pair), an iPSC line derived from a single donor is gene-edited (or treated), to create two iPSC lines that share the same genetic background and only differ at the gene locus of interest (or treatment). Design 2B is an extension of 2A, in which multiple variants (or treatments) are edited into the same genetic background. In Design 3, multiple isogenic pairs are created. The table represents the ability of each design to answer the research questions described on the left. Green: suitable; Orange: possible, but not optimal; Red: not possible.

Different study designs are currently used for iPSC-based disease modelling. Figure 1 outlines the most common designs for different research questions. Design 1 is a design that compares multiple iPSC lines derived from two donor populations (typically patients and controls). Designs 2A & 2B are isogenic comparisons using gene-editing either by introducing genetic variants in standard control lines or by repairing them in e.g., patient-derived iPSCs. Design 2 is also applied in experiments that investigate treatments (e.g., test a compound) in iPSC lines. Design 2A involves a single gene-edit or treatment in one iPSC line. Design 2B expands this to a series of gene-edits or treatments within one iPSC line. Lastly, Design 3 combines these features, comprising a series of isogenic pairs of different (genetically heterogeneous) individuals. Notably, these designs could be extended by including multiple clonal iPSC lines. This could serve to control for unwanted clone-specific aberrations that may occur during reprogramming, gene editing or iPSC passaging. However, using multiple clones inflates the false positive rate, and statistical power benefits are limited [21, 22]. Therefore, the current study focuses on the use of a single clone per individual.

These different designs differ in (1) which types of disorders they can model, (2) to what extent conclusions can be generalized, (3) to what extent they can be applied for personalized medicine, and (4) which data structures they generate and statistical approaches they dictate. First, gene-edited isogenic designs (Designs 2 A&B, 3) can model monogenic disorders, but modelling polygenic disorders is challenging and disorders for which the genetic component is not yet fully elucidated (idiopathic/sporadic disorders) cannot be modelled. Design 1 defines ‘cases’ based on diagnostic status and is therefore suitable for mono- and polygenic disorders as well as idiopathic disorders. Second, Designs 1, 2B, and 3 allow generalization of conclusions to the gene of interest, genetic background, and/or the patient population. Design 2B is uniquely suited to study effects of different genetic variants in one specific gene, but conclusions cannot be drawn beyond the specific genetic background used. Conversely, Design 3 is optimal to investigate the effect of a specific genetic variant in different genetic backgrounds. Designs 1 and 3 can model population-level genetic heterogeneity. Therefore, conclusions can be generalized to the overall patient population. Third, iPSC-based studies are also suited for personalized medicine: especially Designs 2 A&B are suited to test a single (Design 2A) or multiple (Design 2B) therapeutic options in a specific individual patient. Fourth, different designs differ in the way the data are collected. Consequently, data structures differ and this has statistical ramifications (see Supplementary textbox 1&2). Taken together, study designs differ in many dimensions, depending on research question and application. The choice between different designs has drastic consequences for data structure and statistical requirements. To assess how these choices affect statistical power and required sample sizes, a systematic analysis of variation sources and power is indispensable.

### Estimation of the variance contributed by iPSC line and culture batch

To obtain representative estimates of data variation, we quantified variance in real experimental data obtained from three assays commonly used in iPSC-based studies: mass spectrometry proteomics (Fig. S1), morphological analyses using immunocytochemistry (Fig. S2), and synapse physiology using patch-clamp (Fig. S3). Measurements were taken from iPSC-derived neurons from five different individuals (from here on referred to as ‘lines’) and multiple culture batches. In a separate experiment, iPSC-derived neurons that were differentiated by NGN2 expression driven from a ‘Safe Harbour’ locus were recorded using patch-clamp electrophysiology, which allows for controlled dosage of NGN2 expression between neurons (Fig. S3, green boxplots).

Neurons were studied 6 weeks after differentiation. At this time point, neurons had a mean dendrite length of 1107–1311 um, and a mean synapse density of 0.0585–0.1146 synapses per um (Fig. S2). Synapses were functional, showing spontaneous and evoked responses as well as short-term plasticity (Fig. S3). Mass spectrometry proteomics showed similar protein detection in both culture conditions, with 4079 proteins detected in neurons of both conditions, and 97% (4079 out of 4208) of the neuron-only proteins detected in both conditions (Fig. S1C). After filtering for synaptic proteins using SynGO [23], 658 out of 674 (i.e., 98%) of synaptic proteins from neurons were detected in both conditions (Fig. S1D).

Together, these datasets serve as pilot experiments to estimate variance and subsequently perform power analyses to inform future studies. The total variation per parameter was quantified by the coefficient of variation (CoV, Supplementary table 1). A statistical comparison of the CoVs for all measured parameters in neurons induced by lentiviral NGN2 expression or expression from a ‘Safe harbour’ locus, revealed no significant difference in total variation between these iPSC-lines (Fig. S4A). Figure S4 shows the variance measured in the present study together with previously published studies with similar culture methods and experimental readouts for both mouse primary and iPSC-derived neuron datasets (Fig. S4B–F).

**Fig. 2:**
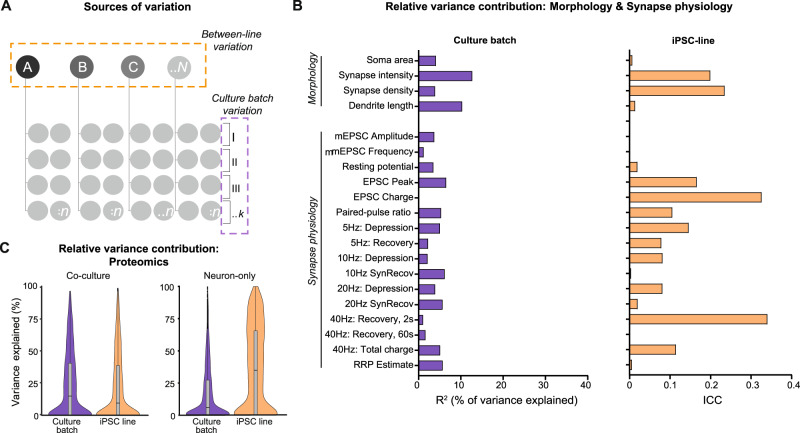
Culture batch and inter-individual variation contribute to the total variance. **A** Schematic overview of the study, indicating sources of variation: inter-individual variation (i.e., variation introduced by including different individuals; orange box) and culture batch variation (i.e., variation introduced by acquiring data from different culture batches; purple box). Note that for this study, five iPSC-derived lines were used for morphological characterization, four lines for electrophysiology, and three lines for proteomic analyses. For the proteomics analysis, neurons were either cultured on a glia feeder layer (‘co-culture’) or on coating without glia (‘neuron-only’). **B** The contribution of different sources of variance is assessed by plotting the proportion of variance explained by culture batch (purple) and line (orange) for each parameter, as calculated following Nakagawa 2017 [17] for details, see Supplementary Methods). Values per parameter are included in Supplementary Table 2. Together, the variance contributed by line and culture batch accounted for a median of 10% (IQR: 6.0–21%) of total variance. **C** The explained variance calculated for all proteins in the proteomics dataset from neuron-glia co-cultures (top violin plot) and neuron-only cultures (bottom violin plot). For co-cultures, median variance explained by culture batch is 14.8% and for line 9.4%. For neuron-only cultures, the median variance explained by culture batch is 5.5% and 34.3% for line.

**Fig. 3:**
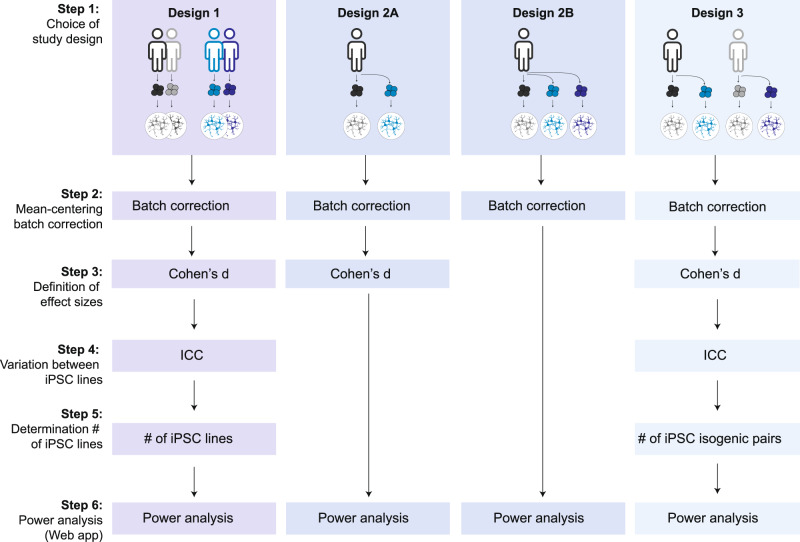
Flow chart for a priori power analysis. Flow chart describing the steps for performing power analysis per study design. Based on pilot data, variance estimates can be obtained and used to calculate effect sizes. For Designs 1 and 3, intra-cluster correlation (ICC) values should be taken into account. Power analyses for a wide range of study design scenarios can be performed using the freely available online application.

We set out to quantify two known sources of variation in human iNeuron studies: batch and line. The variation contributed by multiple “culture batches” (Fig. 2A, B) was estimated as the proportion of variance (R^2^) explained by culture batch [17]. For morphological and synapse physiology parameters, the variance contributed by culture batch varied substantially between parameters, ranging from 0.2 to 13% (Fig. 2B; Supplementary Table 2). For these datasets, glia feeder layers were included which were previously shown to promote neuronal maturation [24–26]. However, glia feeder layers may also increase variance introduced by culture batch. To assess this, neurons cultured with and without glia were compared using proteomics. Indeed, PCA analysis showed that 51% of the variance between the proteomics samples was explained by culture conditions. Moreover, glia feeders increased the variation contributed by culture batch effects: with glia, culture batch R^2^ was 15%, as opposed to 5.5% without (Fig. 2C). Taken together, the different datasets show that including multiple culture batches adds variation to the data (as previously demonstrated: for review, see Volpato and Webber, 2020 [9]) and this source of variation should be considered in statistical analyses (Supplementary textbox 1 and 2) by including culture batch as covariate. In the context of a priori power analyses, culture batch variation can be controlled for by mean-centering the data before using the estimated (unknown) variance to define the expected effect size (Fig. 3, Step 2&3), and including culture batch as a covariate when estimating dependency in the data (Fig. 3, Step 4).

Designs 1 and 3 include data from multiple independent donors as another source of variation. As multiple neurons are derived from the same donor, inclusion of multiple donors introduces dependency in the data, i.e., dependency between data points derived from the same donor (Supplementary textbox 1 and 2). The degree of dependency is expressed as the intra-class or intra-cluster correlation (ICC; Supplementary textbox 1). In our data (Figs. S1–3), the ICC, i.e., the contribution of ‘iPSC line’ to the total explained variance, differed substantially between data types and specific parameters, ranging from 0.0 to 0.35 (Fig. 2B). Depending on the number of observations taken from each iPSC line, even limited dependency can result in inflated false positive rates or lower power [5, 6]. Hence, including the estimated ICC in power analyses is crucial to accurately determine the number of independent iPSC lines to be included to achieve sufficient power (Fig. 3, Step 4&5).

The variance estimates provided here for morphological, electrophysiological, and proteomic parameters can be used as a first guidance for power predictions of future iPSC-based studies. As the contribution of independent iPSC lines and culture batches to the total variance varies considerably between parameters, assay types, and culture conditions, and may additionally vary as a function of e.g., source material, donor characteristics, and reprogramming methodology. Additionally, for certain assay types, correction for multiple testing will affect the alpha level and thus alter the attainable statistical power. Thus, we created a web tool delivering power curves for a wide range of parameter settings. This tool enables iPSC researchers to perform a priori power analyses using pilot data-derived parameter settings (Fig. 3, Step 6) for each of these designs, while accounting for clustering of the data points introduced by using multiple iPSC lines. Additionally, the R-scripts used to perform our power simulations can be downloaded from the web tool, allowing researchers to tweak and add parameters to fit specific experimental circumstances and then perform customized power simulations for all scenarios. In the next sections, examples of power calculations are provided to illustrate the main determinants of statistical power for the four different designs.

### Power analysis for case–control designs

**Fig. 4:**
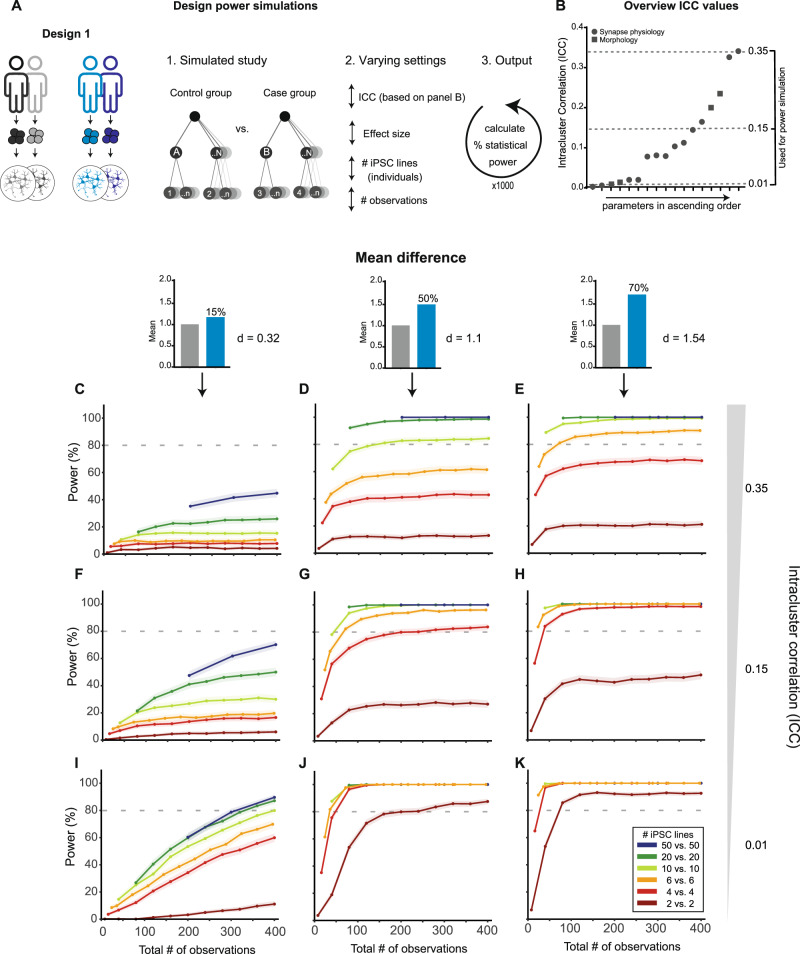
Power simulations to calculate statistical power of Design 1-type studies. **A** Schematic overview of the study design for the power analysis. In this hypothetical scenario, two conditions (control and case) are compared. Within each condition, multiple individuals are sampled. The statistical power is calculated by a simulation experiment (1000 simulations per scenario) varying the number of iPSC lines (either 2, 4, 6, 10, 20 or 50 per group, i.e., 4, 8, 12, 20, 40 or 100 lines in total) and the number of observations per individual. To model a series of scenarios, the simulations were performed for three representative ICC values. **B** ICC values (as shown in Fig. 3B) per parameter sorted in ascending order. Three representative ICC values (reflecting low, medium and high clustering) were selected for the power simulation. **C**–**K** Simulated power curves, showing the relationship between statistical power and the total number of observations (number of iPSC lines times the number of observations per iPSC line).For each plot, the grey dotted line represents the cut-off value of 80% power. To assess statistical power for a range of effect sizes, three scenarios were compared, in which the two groups showed a mean difference of 15% (small), 50% (medium) or 70% (large). Corresponding Cohen’s *d* values were calculated using these mean differences and measured variance of the morphology parameter ‘Synapse Density’ (SD: 0.038) from the data example: 15% mean difference: *d* = 0.32; 50% mean difference: *d* = 1.1; 70% mean difference: *d* = 1.54.

To predict the statistical power for Design 1, we performed power simulations for a typical (hypothetical) case–control study: two experimental groups with *N* number of independent iPSC lines and *n* number of observations per independent iPSC line (Fig. 4A). In a multilevel design, the power to detect mean differences on the dependent variable between cases and controls depends not only on *N*, *n*, and the effect size, but also on the ICC, i.e., the similarity of observations taken from the same iPSC line [5]. To select representative ICC values, the observed (batch-corrected) ICCs from Fig. 3 were sorted in ascending order (Fig. 4B) and three ICCs (low, medium, high) were chosen that cover the observed range (0.01, 0.15, and 0.35). Effect sizes of the mean group difference on the dependent variable were expressed as Cohen’s *d*, which divides the difference between group means by the pooled (batch-corrected) standard deviations. Notably, since effect size Cohen’s *d* depends not only on the mean difference between the two groups but also on the variation in the data, *d* can vary considerably between experimental set ups and parameters. For our power simulations, we selected effect sizes based on our (batch-corrected) morphology data, specifically the parameter ‘Synapse Density’ corresponding to selected mean group differences of 15%, 50 and 70%. All power simulations were subsequently performed using a simplified statistical model that did not include any covariates, i.e., as one would do for data which are corrected for possible covariates like batch effects.

For each simulation scenario, the power to detect a mean difference on the dependent variable between cases and controls was estimated for an increasing number of total observations, where the total number of observations is a function of the number of independent iPSC lines *N* (either 2, 4, 6, 10, 20 or 50 lines per experimental group; Fig. 4C–K), and the number of observations per line *n*. In each graph, the 80% power criterion is indicated, as this is conventionally considered acceptable power.

As expected, simulations generally showed that the lower the ICC and the larger the effect size, the higher the maximum power with the same number of independent iPSC lines *N* and observations *n*. In none of the scenarios, sufficient power was reached to detect a mean difference of 15% based on our data (Cohen’s *d* of 0.32; Fig. 4C, F, I). Additionally, these simulations indicate that studies with only 2 independent iPSC lines per condition (dark red lines in Fig. 4C–K) are bound to fail to detect real effects, except when dealing with (very) large effects and parameters with low ICC values (ICC = 0.01; Fig. 4J, K). For medium to high ICC values, in many instances the power has an asymptote below 100% and thus reaches a point where adding more observations *n* per line does not yield more power. Including more independent iPSC lines (*N*) does, however, increase the maximum attainable power, and sufficient power can be reached to draw generalizable conclusions for disease modelling studies involving genetically heterogeneous iPSC lines. The effect of increasing the number of lines *N* is most noticeable for small effect sizes, but observed for all effect sizes included: sufficient power to detect a medium-sized effect in the context of a high ICC can only be achieved by including a minimum of 10 independent lines per condition, whereas with fewer lines, the power plateaus below 80% (Fig. 4D). Overall, and in line with previous studies (e.g [5]), across all effect sizes and ICC values, the inclusion of more independent iPSC lines *N* increases power more than inclusion of more observations per line *n*. For the same total number of observations (N*n), studies involving more lines consistently have a higher power in all scenarios.

To assess the number of independent iPSC lines generally included in iPSC-neuron case–control studies, we performed a PubMed literature search in high-impact journals (Fig. S5). The number of independent iPSC lines per condition ranged from 1 to 14, with a median of 3 independent iPSC lines per condition (Fig. S5); 75% of the reviewed studies included 4 or less independent iPSC lines per condition. Our power simulations show that the majority of high-impact published iPSC case–control studies have included a lower number of iPSC lines than necessary to meet conventional power requirements.

### Power analysis for isogenic designs

**Fig. 5:**
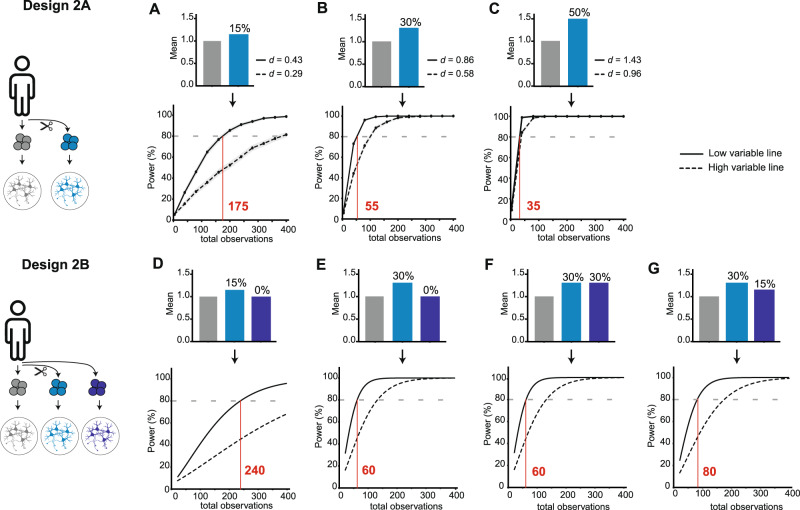
Power simulations to calculate statistical power of Design 2-type studies. **A**–**C** Design 2A describes a comparison within a single isogenic pair. Simulated power curves are shown for three mean difference-scenarios. The corresponding Cohen’s *d* values were calculated for an iPSC-line showing low variability (SD_C2_ = 0.031; thick line) or high variability (SD_C1_ = 0.044; dashed). Corresponding Cohen’s *d* values: 15% mean difference: *d* = 0.29 (high) and 0.43 (low); 30% mean difference: *d* = 0.58 (high) and 0.86 (low); 50% mean difference; *d* = 0.96 (high) and 1.43 (low). **D**–**G** For Design 2B, a hypothetical study was simulated with three experimental groups for the high- and low-variability iPSC lines, as for Design 2A. Four scenarios were tested, comparing the impact of having a small (15%) or medium (30%) mean difference (**D** and **E**), and the impact of including two groups with the same (**F**) or different (**G**) effect sizes.

Next, power simulations were performed for Designs 2 A and B. Similar to Design 1, (batch-corrected) data from synapse density was used to apply realistic parameter settings for our simulations. Since in these designs only a single founder iPSC line is included, simulations were performed based on data both from the line showing highest data variation (i.e., high variable line; C1 in Figs. S1–3) and the line showing lowest variation (i.e., low variable line; C2 in Figs. S1–3). The variance was kept equal between experimental groups, based on the assumption that the variance did not change due to experimental manipulations like the gene editing process. For Design 2A, power was estimated for an increasing number of observations between the two conditions showing a simulated mean difference of 15% (Cohen’s *d* of 0.29 for high and 0.43 for low variable line), 30% (Cohen’s *d* of 0.58 for high and 0.86 for low variable line) and 50% (Cohen’s *d* of 0.96 for high and 1.43 for low variable line) (Fig. 5A–C). As expected, the total number of observations required to reach 80% power decreased when effect sizes increase. For the low variable line, 175 observations were required to achieve 80% power to detect a mean difference of 15% (Cohen’s *d* of 0.43), and 35 observations for a 50% mean difference (Cohen’s *d* of 1.43). The high variable line required higher numbers of observations to reach the 80% power cut-off, especially with smaller effect sizes; 400 observations for a 15% mean difference (Cohen’s *d* = 0.29) and 100 observations for a 30% mean difference (Cohen’s *d* = 0.58). Thus, power analysis in a single isogenic pair design scales with the anticipated mean difference and with the intrinsic variability of the founder iPSC line.

To illustrate the most important power considerations for Design 2B, we performed simulations for a hypothetical design of three conditions (e.g., control and two experimental conditions) showing different combinations of mean effects. For a scenario in which only one experimental condition had a 15% mean difference to control, a total of 240 observations were required for the low variable line (Fig. 5D). This was reduced to 60 observations for a 30% mean difference (Fig. 5E). When both experimental conditions showed a 30% mean difference, the total number of observations remained unchanged at 60 (Fig. 5F). However, if one experimental group had a 15% mean difference, in combination with a 30% mean difference for the other group, the number of required observations increased to 80 (Fig. 5G), which was higher than scenarios described in Fig. 5E and F. Thus, in case of 3 conditions, inclusion of multiple groups that are expected to show the same experimental effect size does not change power, whereas including groups that are expected to show differential effect sizes negatively impacts power to detect an overall effect. As expected, a substantially higher number of observations was required for the high variable line in all scenarios. Thus, required observations to reach 80% power for Design 2B did not only depend on the size of anticipated mean differences and intrinsic iPSC line variability, as in Design 2 A, but also on the pattern of mean differences between the experimental groups.

### Power analysis for multiple isogenic pairs

**Fig. 6:**
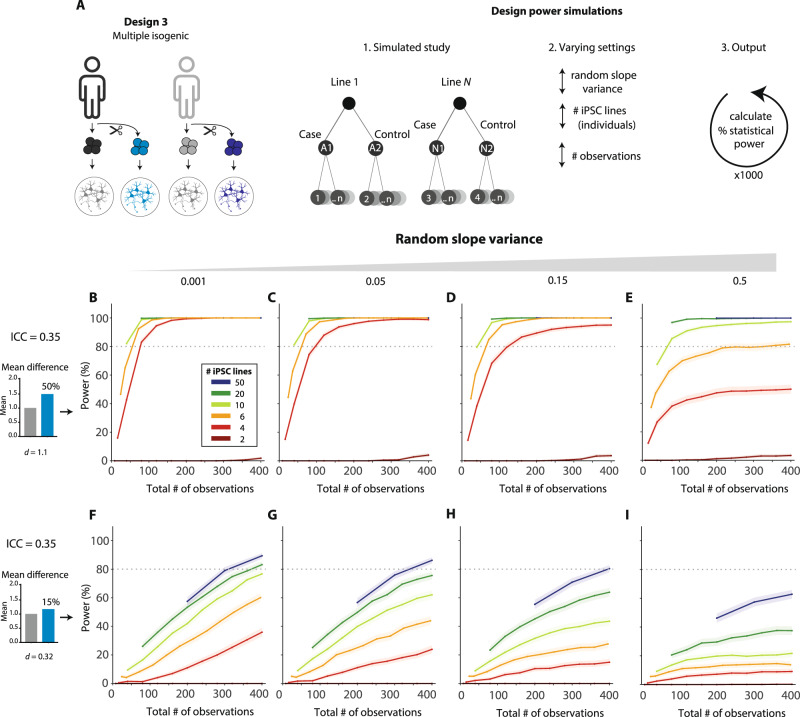
Power simulations to calculate statistical power of Design 3-type studies. **A** Schematic overview of the study design. Several independent iPSC lines are sampled and for each line, an isogenic ‘control’ is generated. Thus, two conditions (‘cases’ and ‘controls’) are compared, while accounting for clustering in the data that is due to the use of multiple individual iPSC lines **B**–**I** The statistical power is calculated by a simulation experiment (1000 simulations per scenario) as for Design 1 (Fig. 4), for the highest ICC value from Fig. 4 (0.35), the high (SD_C1_ = 0.044) and low (SD_C2_ = 0.031) variable lines as for Design 2 A, and two mean difference scenarios (50%: **B**–**E**; 15%: **F**–**I**). In addition, four different slope variance values are tested: 0.001 (negligible); 0.05 (‘medium’; Aarts et al. 2015); 0.15 (‘high’: Aarts et al. 2015); 0.5 (‘extreme’).

As shown above, experiments using isogenic lines (Designs 2 A and 2B) are superior in terms of attainable power to case–control designs (Design 1). However, because these Designs feature only 1 line, the results are limited in terms of generalizability of findings compared to experiments using multiple genetically heterogeneous case and control iPSC lines. For example, the effect of a genetic mutation can differ considerably between individuals as a function of genetic background. As a paired design for multiple isogenic lines, Design 3 combines the benefits of both approaches (Fig. 6A). We performed simulations to assess the power for this study design. Simulation parameters were selected that were previously used for the power simulations for Design 1: a high ICC value (0.35), and either a medium (50% mean difference based on our dataset; Cohen’s *d* = 1.1) or small (15% mean difference in our dataset; Cohen’s *d* = 0.32) effect size. Besides estimation of the main effect of the introduction or repair of the genetic mutation on the outcome variable, Design 3 allows the possibility to assess whether the effect of the genetic mutation is the same in all lines, or varies as a function of genetic background. Consequently, an additional variance parameter can be estimated in this type of design: the ‘slope variance’ (see Supplementary textbox 2), i.e., variance in the effect of ‘Condition’ between different isogenic pairs. To illustrate the effect of this variance parameter, four values were included: a very small (almost negligible) variance (0.001); a small (0.05) and a large (0.15) value, as previously used by (Aarts et al., [6]) based on guidelines of (Raudenbush and Liu, 2000 [27]), and an extreme value (0.5). Simulation showed that for Design 3, much higher power is achieved compared to Design 1 with a limited number of iPSC lines. For instance, to detect a Cohen’s *d* of 1.1, 80% power can be achieved with 4 isogenic pairs even if the slope variance is considerable (Fig. 6B–D), whereas power reaches a plateau under the same input conditions in a Design 1 situation even when a total of 12 lines (6 vs 6; Fig. 4D) is used. Thus, using multiple isogenic pairs considerably improves statistical power compared to a case–control design, limiting the number of lines required to detect true differences. However, it should be noted that this design does not improve power endlessly: to detect small differences (e.g., a mean difference of 15%; Fig. 6F–I), power still reaches a plateau, or a large number of observations is required to reach sufficient power. Moreover, extreme values of slope variance compromise power substantially. However, such extreme slope variation would likely compromise the interpretability of the experiment as a whole, as it implies extreme differences in the effects of the mutation in different genetic backgrounds. Our web application includes a wider range of scenarios, paralleling the ICC values and effect sizes also assessed for Design 1.

### Statistical power considerations

Together, the power simulations in Figs. 4–6 illustrate several general conclusions on the impact of different factors on statistical power in iPSC-based disease modelling. First, of course the bigger an effect size, the lower the number of independent iPSC-lines and total observations needed to reach sufficient statistical power. Second, including multiple iPSC-lines leads to dependency in the data and results in a “power-plateau”: a point in the power curve where adding more observations from the same lines does not increase power (Fig. 4C–K). Instead, increasing the number of independent iPSC-lines does increase the maximum attainable power. Third, lower levels of dependency in the data (low ICC) support a higher maximum attainable power with the same number of iPSC-lines *N* and observations *n* (Fig. 4C–K). Importantly, including more lines increases not only the statistical power to detect the experimental effect, but also the generalizability of the results (Fig. 1). In contrast, in isogenic designs only one founder iPSC-line is included. In this design, increasing the number of total observations will increase statistical power but generalization of findings is limited (Figs. 5A–C; 1). Fourth, in isogenic designs, within-line variation should be taken into account because higher within-line variability results in lower statistical power for the same mean difference and sample size (Fig. 5A–G). Fifth, for isogenic series, the statistical power is affected by the pattern of means: inclusion of multiple groups that are expected to show the same experimental effect size does not affect power, whereas including groups with varying effect sizes negatively impacts power to detect an overall effect (Fig. 5D–G). Sixth, when using multiple isogenic pairs, the variance in the effect of the experimental manipulation between different isogenic pairs, such as gene editing, affects the statistical power. The lower this random slope variance and the higher the effect size, the higher the maximum power with the same number of independent iPSC lines *N* and observations *n* (Fig. 6B–I). Lastly, for the same ICC, number of observations, and effect size, using multiple isogenic pairs results in substantially higher statistical power then using a case–control design (Figs. 4, 6). Although the power simulations presented here only cover a limited number of scenarios, these concepts are true for all possible combinations of settings. Our online tool can be used to explore and visualize these concepts for a wide range of scenarios.

## Discussion

In this study, we compared commonly used study designs for iPSC-based disease modelling in terms of applicability for different research questions, statistical analysis, and attainable power. Variance estimates used in our power simulations were based on original data from five independent healthy control lines for immunocytochemistry, electrophysiology and proteomics. The quantified variance was used to define the power analysis settings by calculating effect sizes after mean-centering the data to correct for culture batch variation (Fig. 3), determining a representative range of ICCs for power analyses (Fig. 4B), calculating Cohen’s D’s corresponding to selected mean-differences based on one example parameter (namely, synapse density; Figs. 4–6), and to determine representative within-line variances shown in Fig. 5.

Isogenic designs are very suitable to study effects of a particular gene variant (Design 2A), or variants (Design 2B), while maintaining the rest of the genetic background identical between conditions, thus minimizing unsystematic variation and optimizing the statistical power. These isogenic designs are especially suited for, e.g., personalized medicine approaches, aimed at finding customized treatments for individual patients in a clinical setting. Moreover, a study based on isogenic lines may provide important first insights into potentially relevant disease mechanisms that may generalize to the broader patient population. As such, isogenic experiments may serve as a case study to inform subsequent studies aiming to generalize the conclusions to the patient population. However, effects observed in a single genetic background may not generalize to other circumstances. In order to generalize to a patient group or population, inter-individual variation needs to be incorporated in the study design.

Case–control study designs (Design 1) are suited for this purpose, yet the clustering of data points introduced by obtaining multiple measurements from different genetically heterogeneous individuals has a considerable impact on the attainable statistical power. Previously, gene expression studies indicated that donor-specific differences account for a considerable proportion of the overall variability [21, 28–34]. Consequently, several studies conclude that iPSC-based studies benefit from including more donors, rather than more clonal iPSC lines from the same individual [21, 22, 29, 31, 35, 36]. Here, we extend the characterization of variance contributions to functional synaptic readouts and provide a quantification of the consequences of inter-individual variance on statistical power for a range of scenarios and study designs. In line with previous studies [22, 36], we conclude that iPSC-based studies generally require a high number of independent iPSC lines to observe true effects. If the number of independent lines available is limited, there is a constraint on the maximum effect size that can be reliably detected. Importantly, adding more observations from a limited number of independent lines does not improve statistical power beyond a certain point. This observation has important ramifications for the feasibility of iPSC studies, since the addition of more independent iPSC lines is typically (much) more costly and (much) more difficult to realize than adding more observations per iPSC line. In this light, analysing a series of isogenic pairs (Design 3) within a study may prove an attractive novel approach. Because in this design, the experimental effect is assessed within each iPSC line, the variance between the iPSC lines does not impact the power whereas it does for Design 1 studies, where the experimental effect is assessed between iPSC lines. Thus, Design 3 capitalizes on the use of isogenic lines to reduce variation, thus improving power on the one hand, while including multiple genetic backgrounds to facilitate generalization on the other. Taken together, iPSC studies can answer a range of research questions and selection of the optimal study design is key to optimizing the scientific impact of this technique.

The design considerations outlined in this paper and the web tool and available R-scripts for a priori power estimation will help researchers to choose the optimal design and statistical analysis tailored to their research questions. Whilst the conclusions of this study are tailored towards iPSC-based brain disease modelling studies, the considerations regarding dependency in data and statistical power also apply to other, non-iPSC-based experimental designs, like experiments in vivo or in primary neuron cultures. Performing a priori power analysis increases validity and reproducibility of future experiments involving iPSC-derived neurons and as such help realize the potential of this new technique. The settings used for the power simulations presented here, were based on variance analysis of several example data types obtained from a set of five heterogeneous iPSC lines. Experiments using other data types or different cell types may yield different statistical power, and thus may require researchers to perform their own pilot studies to inform their study design. Practical constraints may exist to the statistical power that can be achieved. For instance, the use of a large number of iPSC lines or the editing of multiple mutations may be challenging due to limited resources (time, funding) or availability of iPSC lines. As the field progresses, optimization of culturing and differentiation protocols, customized matching of iPSC lines (on e.g., source material, passage number, reprogramming method, donor age, sex, ancestry) and implementation of standard operating procedures, including quality controls, may reduce variation within and between iPSC lines, thus improving attainable power. In addition, accessibility of iPSC lines is increasingly facilitated by biobanking initiatives, rendering large numbers of well-characterized, high-quality iPSC lines widely accessible, facilitating researchers to use iPSC lines that are optimally tailored to their research questions. Such improvements are expected to reduce data variability and increase scalability of iPSC-neuron experiments. Together with rigorous study designs and appropriate statistical analyses, iPSC technology can significantly advance scientific progress in brain disorder modelling.

### Limitations of the study

To inspire the power simulation settings in this study, we selected five iPSC lines that differ in terms of source material, reprogramming methodology and donor characteristics. More elaborate experimental set-ups may include sets of donor iPSC lines matched on these characteristics or characterize the contribution of these different factors to overall power, or may involve a larger number of iPSC lines. Nevertheless, the selected sample set represents a likely scenario for the modelling of rare diseases, for which access to patient donors may be limited, requiring researchers to use previously sampled materials. The datasets used here serve as a first guidance to estimate the relative variance contributions, but after quantifying variance explained by culture batch and line combined, more than 50% of the variance in the data still remained unexplained. Therefore, additional covariates could be identified and project-specific optimization steps may be undertaken to reduce overall and between-line variance, thus increasing statistical power. A wider range of scenarios, including scenarios with lower variance, is represented in our web tool. The power analyses in this study are based on the specified statistical models, and assume data are corrected for covariates and collected in a balanced fashion (i.e., acquiring equal numbers of observations per iPSC-line). To allow researchers to assess the attainable power for less frequently used study designs, or for parameter settings different from the ones represented in our web tool (e.g., different alpha-levels), the R scripts used for our power simulations can be downloaded and customized.

## Supplementary information

Supplemental Figures

Supplementary Methods

Supplemental table 2: R2 values (proportion of variance explained), calculated as described in Methods section.

Supplemental Table 1: Coefficient of variation (CoV) values

Supplemental textbox 1. Statistical terms

Supplemental textbox 2. Data structures and appropriate statistical model

## Data Availability

The datasets (proteomics; confocal imaging; electrophysiology) are available from the lead contact upon request.

## Code availability

The Matlab code used to analyse morphological data is publicly available via https://www.johanneshjorth.se/files/SynD/ [16]. The in-house developed Matlab code used for analysis of electrophysiological data and for the power simulations is available upon request. MS-DAP 0.2.6.4 used for proteomics differential expression analysis (DEA) is available at https://github.com/ftwkoopmans/msdap. The R code used to perform the power curve simulations are available upon request. The web app, including annotated scripts, described in this paper can be found at https://jessiebrunner.shinyapps.io/App_PowerCurves/.
