# Supplementary material for: Power and optimal study design in iPSC-based brain disease modelling
Source: Mol Psychiatry. 2022 Nov 16;28(4):1545–56. doi: 10.1038/s41380-022-01866-3 (PMC10208961; doi:10.1038/s41380-022-01866-3)
Supplement: Supplementary file 1 — Supplemental Figures [file 41380_2022_1866_MOESM1_ESM.pdf]

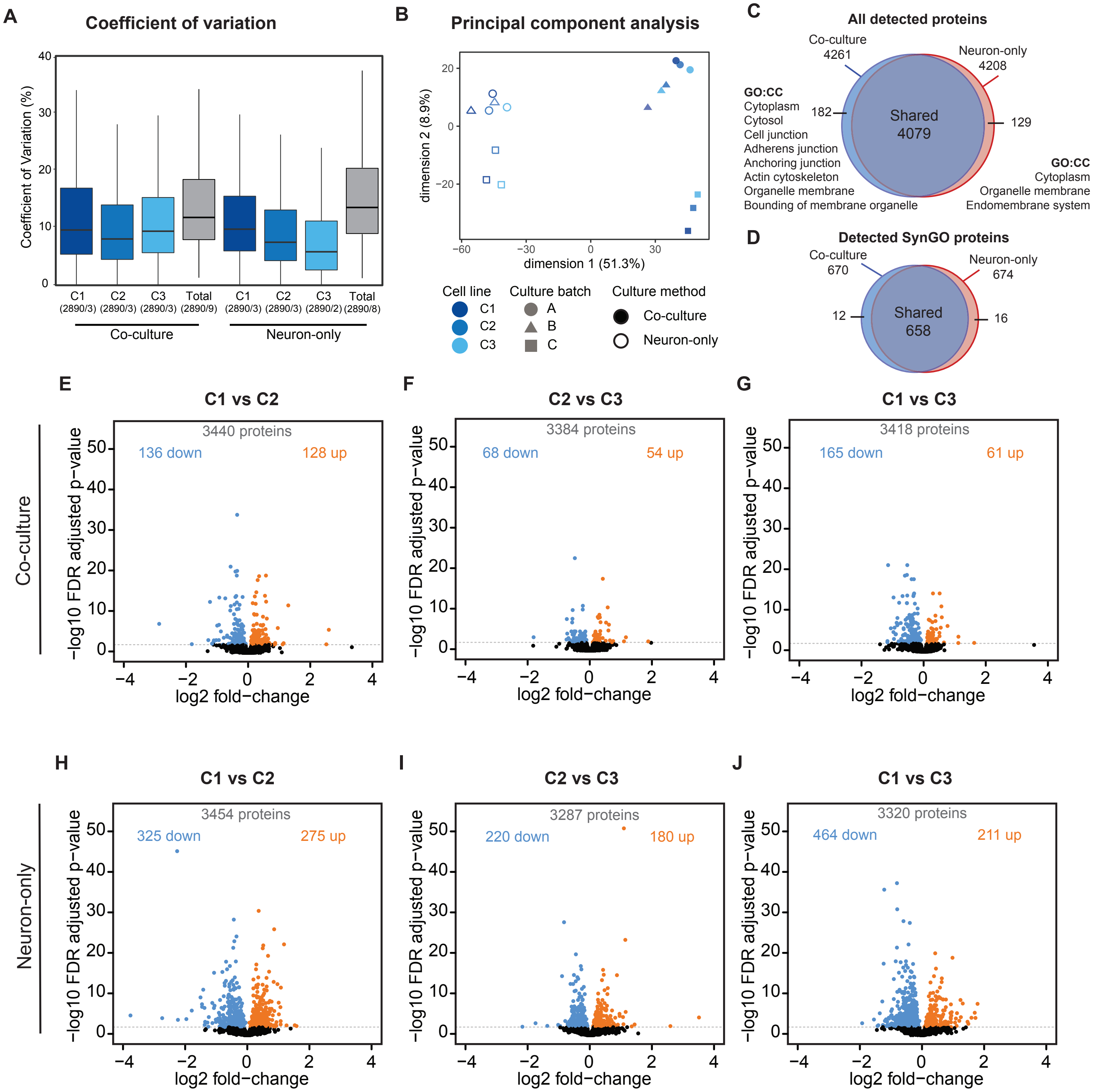

Fig. S1: Variation is observed in the proteome of iNeurons from three independent iPSC lines. Related to Figure 2.
(A) Coefficient of variation (CoV, standard deviation divided by mean) is calculated for protein-level data of each cell-line in each culture condition. Co-culture: C1 (median = 9.74), C2 (median = 8.29), and C3 (median = 9.57). Neuron-only: C1 (median = 9.99), C2 (median = 7.63), and C3 (median = 5.91). (B) Principal component analysis (PCA) score plot of the two first principal components of the peptide intensity of peptides identified in all samples. In total, 24332 unique peptides from 4054 unique proteins were detected in line C1, 23746 peptides from 4010 proteins in line C2, and 23586 peptides from 3999 proteins in line C3. Colour represents iPSC line, shape represents culture batch (biological replicates), fill represents culture method. (C) Overlap between the proteomes of iNeurons cultured with (Co-culture) or without (Neuron-only) rat glia. In total, 4079 proteins were detected in both culture methods: 95% (4079 out of 4261) of the proteins detected in the co-culture condition were also detected in the neuron-only samples. Significantly over-represented Cellular Component (CC) GO annotations in the uniquely detected subsets are listed. (D) After filtering for SynGO annotated proteins, 658 out of 674, 98%, of proteins in the co-culture were also detected in the neuron-only samples. (E-J) Differential abundance analysis was performed to visualize the differences in protein expression levels between different control lines (E) Volcano plot of pairwise comparison between C1 and C2 co-culture. 264 out of 3440 proteins (7.67%) are significantly regulated (q-value > 0.01) with a mean fold change of 1.32. (F) Volcano plot of pairwise comparison between C2 and C3 co-culture. 122 out of 3384 (3.61%) are significantly regulated (q-value > 0.01) with a mean fold change of 1.28. (G) Volcano plot of pairwise comparison between C1 and C3 co-culture. 226 out of 3418 (6.61%) are significantly regulated (q-value > 0.01) with a mean fold change of 1.31. (H) Volcano plot of pairwise comparison between C1 and C2 neuron-only. 600 out of 3454 proteins (17.4%) are significantly regulated (q-value > 0.01) with a mean fold change of 1.37. (I) Volcano plot of pairwise comparison between C2 and C3 neuron-only. 400 out of 3287 (12.17%) are significantly regulated (q-value > 0.01) with a mean fold change of 1.29. (J) Volcano plot of pairwise comparison between C1 and C3 neuron-only. 675 out of 3320 (20.33%) are significantly regulated (q-value > 0.01) with a mean fold change of 1.36.

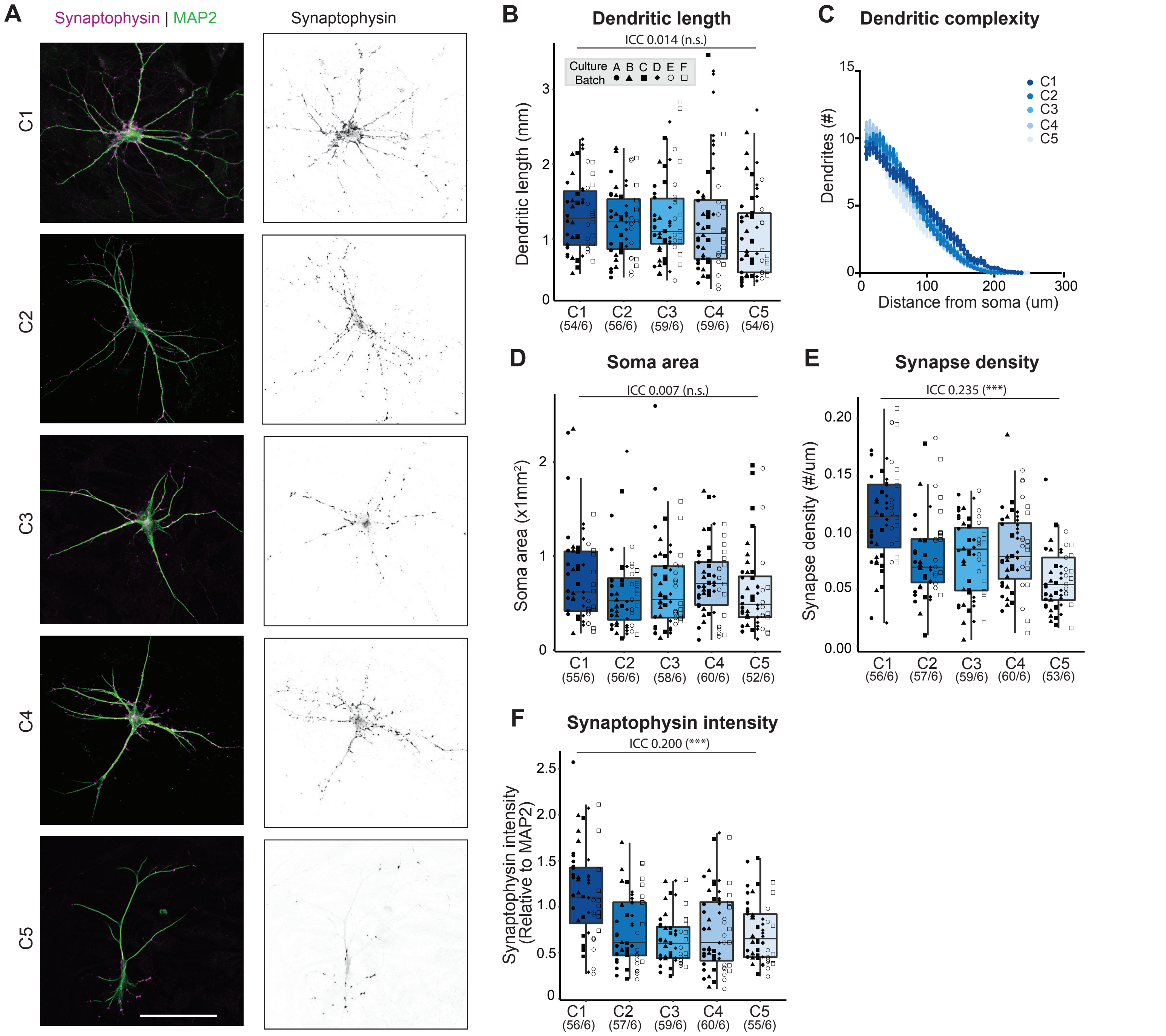

Fig. S2: Between-line variation is observed for synaptic morphology parameters. Related to Figure 2.
(A) Typical examples of all five iPSC-derived iNeurons, stained for dendritic marker MAP2 and presynaptic marker Synaptophysin. A total of 287 iNeurons were assessed for neuronal and synapse morphology parameters from five lines and a total of six culture batches. Scale bar is 100 µm. (B) Dendritic length is comparable between the five lines (ICC=0.014, p=0.176). Model including culture batch as covariate fit significantly better (p=7.3*10-6***). (C) Dendritic complexity, assessed using a Sholl analysis, is similar between the five lines. (D) Soma area is similar between the five lines (ICC=0.007, p=0.310). Model including culture batch as covariate fit significantly better (p=0.034*). (E) Synapse density, i.e. synapse number divided by dendritic length, varies significantly between the five lines (ICC=0.235, p=3.76*10-13). Model including culture batch as covariate fit significantly better (p=0.01*). (F) Synaptophysin intensity varies significantly between the five lines (ICC=0.200, p=1.70*10-12). Model including culture batch as covariate fit significantly better (p=1.4*10-9***).
Data are present in Tukey boxplots. *** = p<0.001; ** = p<0.01; * = p<0.05. Colour represents iPSC line, shape represents culture batch. Sample sizes are indicated on the graphs. Mean and SD for all parameters per group are listed in Table 3.

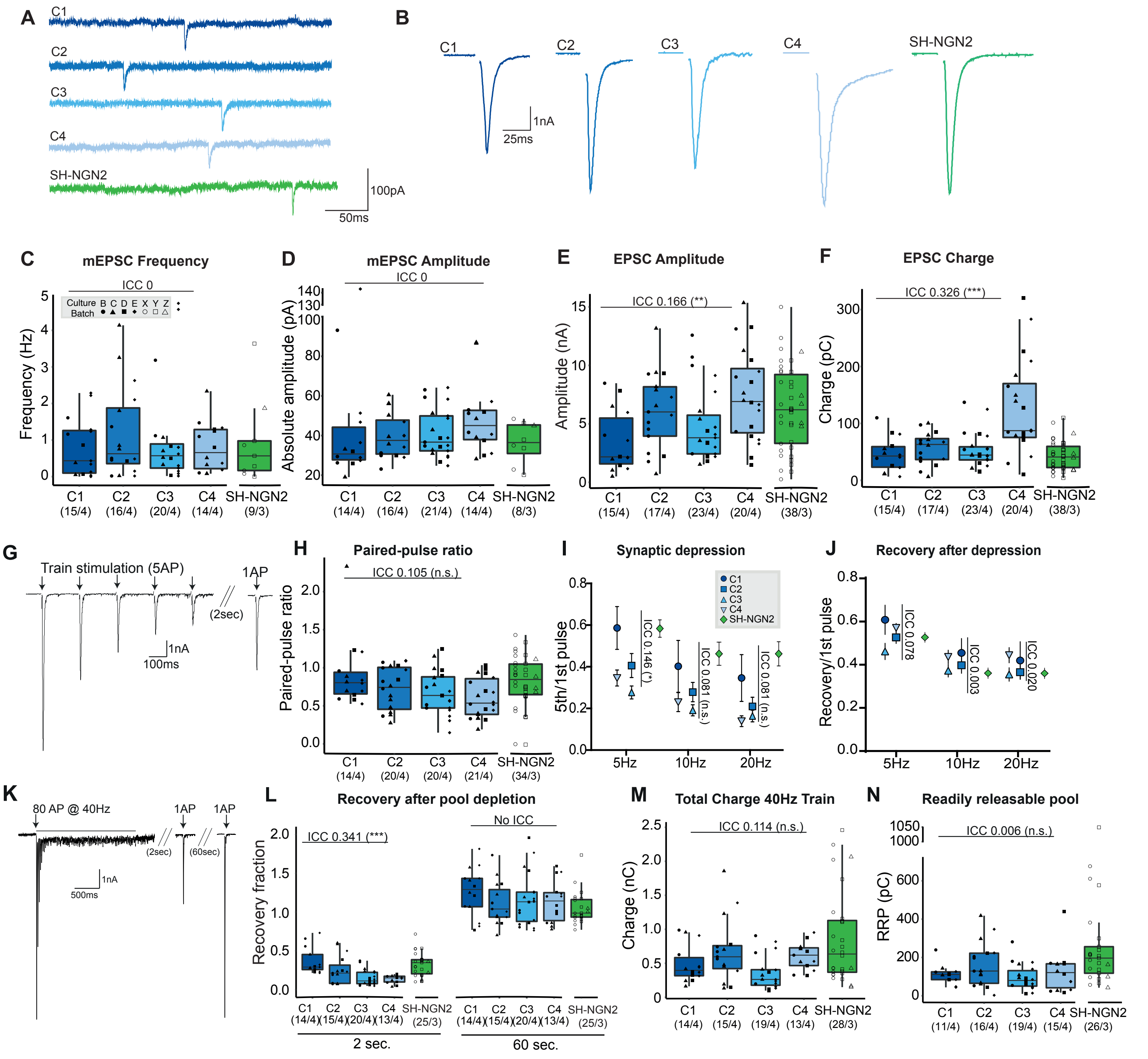

Fig. S3: Between-line variation is observed for several synapse physiology parameters. Related to Figure 2.
(A) Typical examples of spontaneous synaptic activity for each of the five iNeuron lines, measured in voltage-clamp mode. Lines C1-C4 were recorded in parallel, whereas the Safe Harbour-NGN2 line (SH-NGN2; green) was measured in a separate set of experiments. (B) Typical examples of evoked EPSC response to a single action potential. (C) No significant variation in mEPSC frequency between different lines (ICC=0; p=1.00). Because SH-NGN2 iNeurons were recorded in separate experiments, values are plotted in a separated boxplot and not included in the statistical comparison of lines C1-4. (D) No significant variation in mEPSC amplitude between different lines (ICC=0; p=1.00). (E) Peak amplitude of evoked EPSC shows significant inter-individual variation (ICC=0.166, p=0.0088). The fit of a model including culture batch as a covariate does not improve significantly over the base mode(p=0.13). (F) Total charge of evoked EPSC varies significantly between individuals (ICC=0.326, p=3.69*10-5). The fit of a model including culture batch as a covariate does not improve significantly over the base mode (p=0.98). (G) Typical example of a short train stimulation of 5 action potentials at 5Hz, followed by a single 'recovery pulse' 2 seconds after the end of the train. (H) Paired-pulse ratio between individual iNeuron lines is similar (ICC=0.105, p=0.053). The fit of a model including culture batch as a covariate does not improve significantly over the base model (p=0.22). (I) Synaptic depression is defined as the amplitude of the final (5th) EPSC response in the train divided by the amplitude of the first response for each cell. Graph shows the synaptic depression ratios (mean ± SEM) per iNeuron line for the 5Hz, 10Hz and 20Hz stimulation protocols, respectively. Significant variation between lines is observed at the 5Hz stimulation (ICC=0.146, p=0.0253), but not at 10Hz (ICC=0.081, p=0.156) or 20Hz (ICC=0.081, p=0.136). The fit of a model including culture batch as a covariate does not improve significantly over the base mode for each of the three stimulation paradigms (5Hz: p=0.24, 10Hz: p=0.72, 20Hz: p=0.43). Sample sizes (observations/number of independent culture batches): 5Hz: C1 (15/4), C2 (16/4), C3 (21/4), C4 (18/4); 10Hz: C1 (11/4), C2 (14/4), C3 (19/4), C4 (16/4); 20Hz: C1 (13/4), C2 (14/4), C3 (20/4), C4 (16/4). (J) Recovery after depression is defined as the amplitude of the response to the recovery stimulation (2 seconds following the train) divided by the amplitude of the first response of the train. Graph shows the recovery ratios (mean ± SEM) per iNeuron line for the 5Hz, 10Hz and 20Hz stimulation protocols respectively. Variation between lines is not significant after 5Hz (ICC=0.078, p=0.104), 10Hz (ICC=0.003, p=0.475) or 20Hz (ICC=0.020, p=0.368). The fit of a model including culture batch as a covariate does not improve significantly over the base mode for each of the three stimulation paradigms (5Hz: p=0.65, 10Hz: p=0.27, 20Hz: p=0.31). Sample sizes (observations/number of independent culture batches): 5Hz: C1 (15/4), C2 (16/4), C3 (21/4), C4 (18/4); 10Hz: C1 (11/4), C2 (14/4), C3 (19/4), C4 (15/4); 20Hz: C1 (21/4), C2 (14/4), C3 (20/4), C4 (15/4). (K) Typical example of a high-frequency stimulation protocol applied to fully deplete the readily releasable pool. 80 action potentials are delivered at 40Hz frequency, followed by a single stimulation at 2 seconds post-train and one stimulation pulse at 60 seconds post-train, to assess recovery of the response following pool depletion. (L) Recovery fraction after 2 and 60 seconds post-40Hz train are plotted per line. At 2 seconds after the train, significant variation in recovery fraction is observed (ICC=0.341, p=0.00023). After 60 seconds, all iNeuron lines show full recovery and differences between lines do not explain any variation in the dataset (ICC=0; p=1.00). The fit of a model including culture batch as a covariate does not improve significantly over the base mode for recovery after 2 seconds (p=0.80) while no fit was obtained for the 60 seconds-recovery parameter as the conditional ICC was 0. (M) The total charge transferred during the pool-depleting 40Hz train does not vary significantly between lines (ICC=0.114, p=0.0565). The fit of a model including culture batch as a covariate does not improve significantly over the base mode (p=0.29). (N) Back-extrapolation on the last 20 pulses of the 40Hz train to estimate the size of the readily releasable pool, showing no significant differences between iNeuron lines (ICC=0.006, p=0.459). The fit of a model including culture batch as a covariate does not improve significantly over the base mode (p=0.30). A Levene's test indicated equal variances for each parameter tested. Data are present in Tukey boxplots. *** = p<0.001; ** = p<0.01; * = p<0.05. Colour represents iPSC line, shape represents culture batch. Sample sizes are indicated on the graphs. Mean and SD for all parameters per group are listed in Table 3. Note that SH-NGN2 iNeurons were measured in a separate series of experiments, thus not statistically evaluated together with C1-C4 and plotted in a separated boxplot, to allow visual comparison.

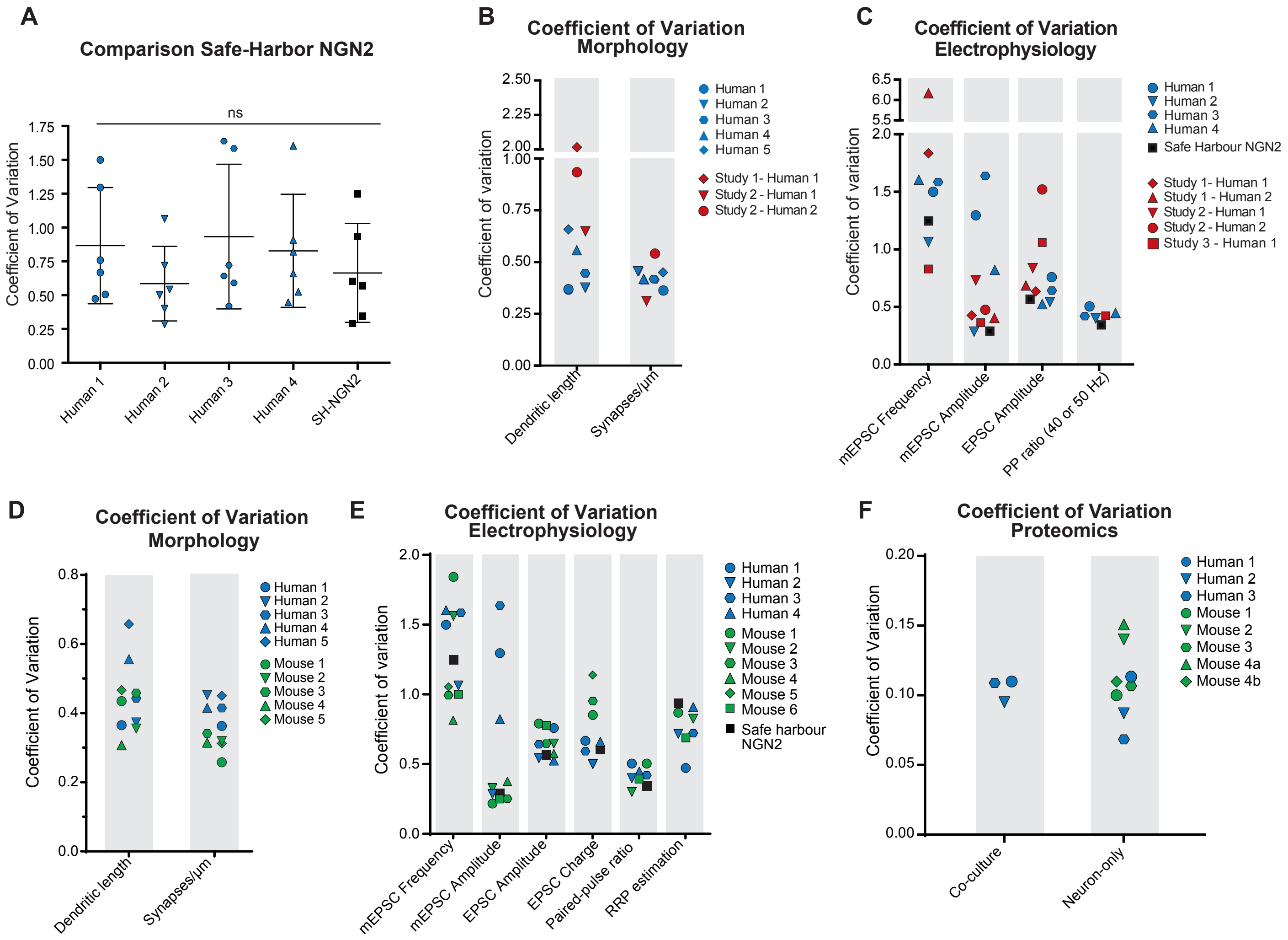

Fig. S4: Human iNeuron variation is comparable to primary mouse neurons and previously published iNeuron studies. Related to Figure 2 and Table 1.

The total variation for parameters in the human iNeuron datasets used in this article was quantified by the coefficient of variation (CoV) per parameter, then compared to CoV values from the safe-harbor NGN2 line measured in this study (A), and to CoV values for similar parameters in previously published iNeuron (panels B&C) and primary mouse neuron (panels D-F) studies. For all calculated CoV values, see Table 1.

(A) CoV values for the electrophysiology measures in Fig. S3 plotted per line. A Kruskall-Wallis ANOVA on the median CoVs revealed no significant difference in total variation between these iPSC-lines.

(B) CoV values for the two morphological parameters for which comparable data were available. Study 1: Meijer et al. (2019), Study 2: Fenske et al. (2019), Study 3: Rhee et al. (2019). (C) CoV values for the synapse physiology parameters for which comparable data were available. Study 1: Meijer et al. (2019), Study 2: Fenske et al. (2019), Study 3: Rhee et al. (2019).

(D) CoV for morphological parameters in figure S3 (blue shapes) compared to CoV values of several published mouse neuron datasets (green shapes; mouse 1, Lammertse, van Berkel et al. 2019; mouse 2, Schmitz et al. (2016); mouse 3, Wierda et al. 2007; mouse 4, Emperador-Melero et al., 2018; mouse 5 (Classen et al. (2020). (E) CoV calculated for electrophysiological parameters in figure S3, compared to CoV values of several published mouse neuron datasets (green: mouse 1, Lammertse, van Berkel et al. 2019; mouse 2, Meijer et al. 2015; mouse 3, Meijer et al. 2017; mouse 4, Wierda et al. 2007; mouse 5, Emperador-Melero et al. 2018; mouse 6, Classen et al. 2020). Total variation was not inherently larger for the iNeuron lines induced by lentiviral NGN2 overexpression compared to an iNeuron line expressing NGN2 from a 'safe harbour'-locus (Fig. S4B, black squares). (F) The median coefficient of variation across all detected proteins is calculated for each iNeuron line (blue shapes) and compared to several proteomics datasets on primary mouse neurons (red shapes; mouse 1, He et al. 2017; mouse 2, Vazquez-Sanchez 2020; mouse 3, Rosato et al. 2019; Mouse 4a, van Oostrum et al. 2020 median CoV across all DIVs; mouse 4b, van Oostrum et al. (2020) median CoV across three conditions).

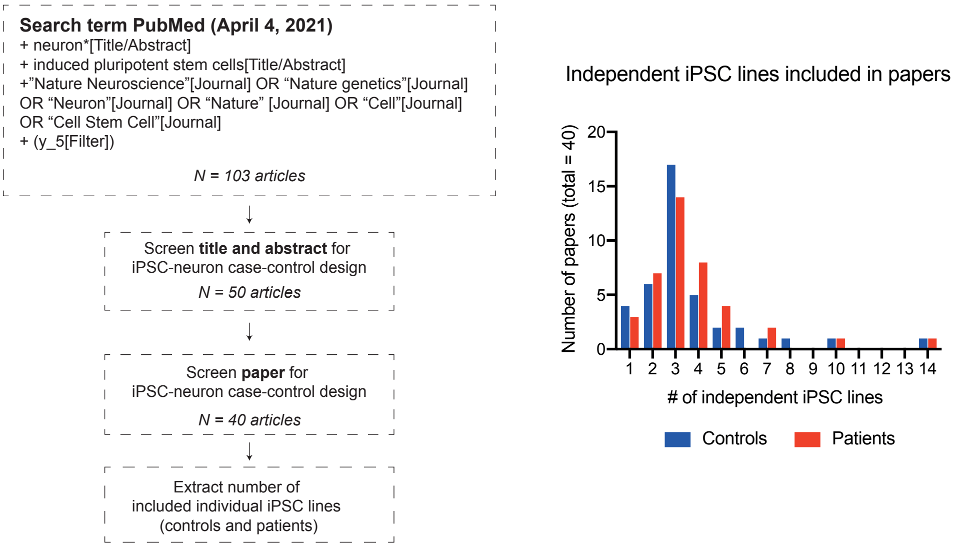

Fig. S5: Literature overview of number of iPSC-lines in case-control studies.
A PubMed literature search was performed to provide an up-to-date overview of the number of iPSC lines high-impact papers generally include. The search term included the words neuron* (title/abstract) and induced pluripotent stem cells (title/abstract), in papers published in the journals Nature Neuroscience, Nature Genetics, Neuron, Nature, Cell or Cell Stem Cell in the last 5 years. This resulted in 103 papers. Next, the title and abstract were screened for case-control designs, which excluded 53 papers. Last, the full text was screened for case-cotrol designs, excluding another 10 papers. The number of iPSC lines/individuals were extracted from the final list of 40 papers. The frequency histogram shows the number of iPSC lines included in these papers, split by 'Control' and 'Patient' lines.

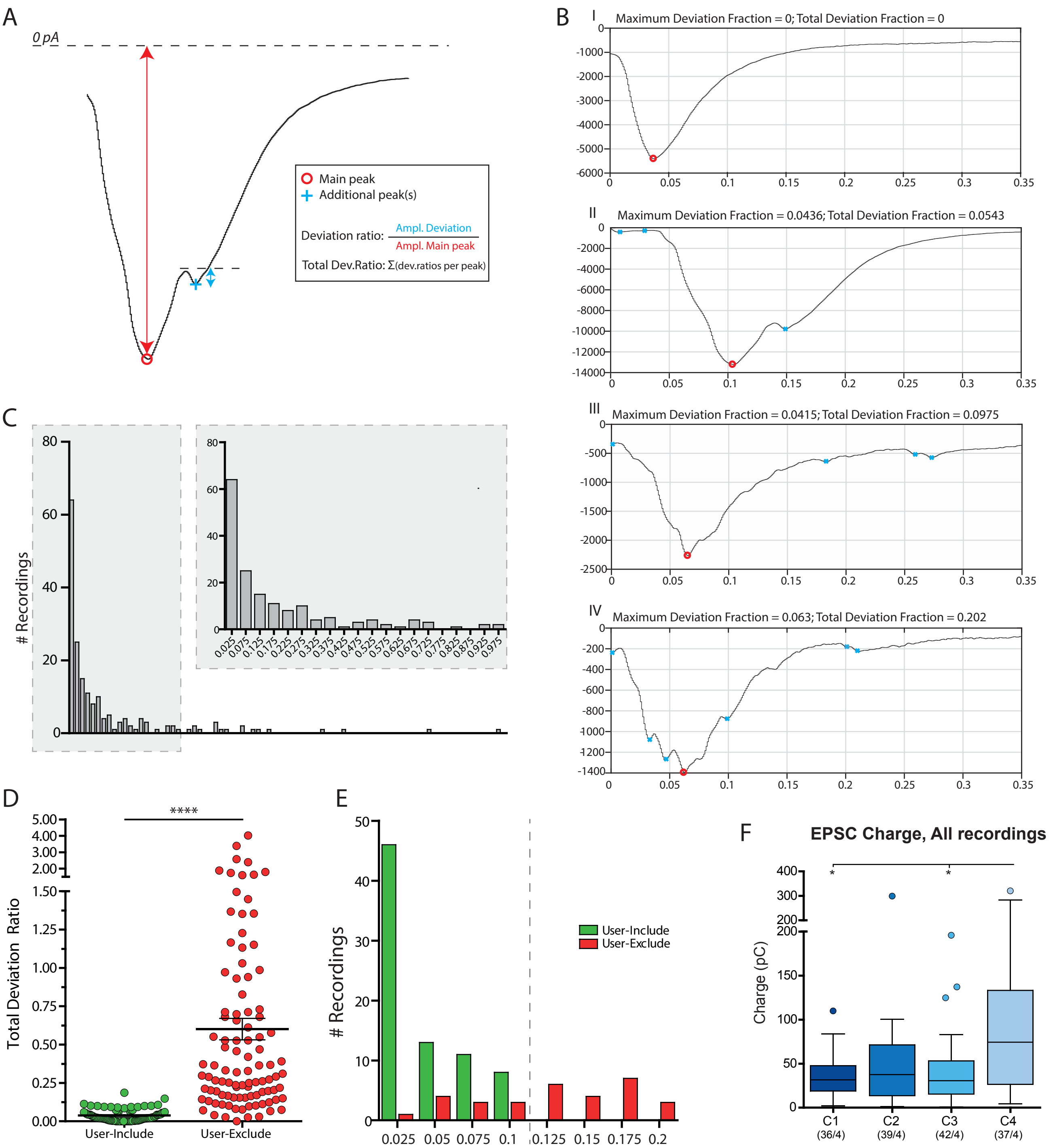

Fig. S6 Algorithm-based selection of 'typical' evoked synaptic responses for subsequent analysis.
(A) Schematic representation of the algorithm detection method. One main peak (red circle) is detected, additional peaks (with a threshold of 20pA minimum) are detected as well (blue crosses). The deviation ratio is taken of the amplitude of the 'deviation' of an extra peak and the amplitude of the main peak. Total deviation ratio is the sum of the deviation ratios calculated for each detected additional peak. (B) Typical examples of algorithm output, showing EPSC responses with a TDR of 0, ~0.05, ~0.1 and ~0.2. (C) Histogram (bin size 0.05; bin center 0.025) of the total deviation ratio (TDR) as quantified by the algorithm for all first evoked recordings from autaptic iNeurons in the dataset, regardless of experimental group. Inset shows a zoom (as indicated by grey box) of the recordings with smallest TDR values, showing the majority of responses has a very smal TDR but a smaller proportion showing a much larger deviation. (D) TDR plotted for recordings that were judged by the user as 'to be included' (User-Include; green) or 'to be excluded' (User-Exclude; red). The TDR values are significantly larger for 'User-Exclude' recordings than for 'User-Include' recordings (User-Include: median 0.0227, User-Exclude: median 0.315; $p < 0.0001$, Mann-Whitney U test). (E) Histogram with the same parameters as panel A, but only including the recordings with lowest TD values and split per user decision. A cut-off value of 0.1 as maximum acceptable TDR was chosen, since this adequately captures all recordings judged as 'User-include', while for larger TDR values all recordings were judged as 'User-Exclude'. (F) Total charge of evoked EPSC of all recordings.
