# Supplementary material for: Power and optimal study design in iPSC-based brain disease modelling
Source: Mol Psychiatry. 2022 Nov 16;28(4):1545–56. doi: 10.1038/s41380-022-01866-3 (PMC10208961; doi:10.1038/s41380-022-01866-3)
Supplement: Supplementary file 2 — Supplementary Methods [file 41380_2022_1866_MOESM2_ESM.docx]

# **Supplemental Materials And Methods**

## **Resource availability**

### **Lead contacts**

For further information and requests for resources and reagents, please contact the Lead Contacts, Matthijs Verhage ([m.verhage@vu.nl](mailto:m.verhage@vu.nl)) and Sophie van der Sluis ([s.vander.sluis@vu.nl](mailto:s.vander.sluis@vu.nl)).

### **Materials availability**

This study did not generate new unique reagents. Plasmids can be obtained from Addgene (refer to Key Resources table for identifier). Induced pluripotent stem cells (iPSC) lines are commercially available or can be obtained upon request by contacting the Lead Contact.

### **Data and Code Availability**

The datasets (proteomics; confocal imaging; electrophysiology) generated during this study are available from the lead contact upon request. The Matlab code used to analyse morphological data is publicly available via <https://www.johanneshjorth.se/files/SynD/> (Schmitz et al., 2011) the in-house developed Matlab code used for analysis of electrophysiological data and for the power simulations is available upon request. The R code used to perform the power curve simulations are available upon request. The web app, including annotated scripts, described in this paper can be found at <https://jessiebrunner.shinyapps.io/App_PowerCurves/>.

## **Experimental models and subject details**

### **Laboratory animals**

Glia were prepared from newborn P0-P1 pups from female Wister rats (Crl:WI, strain code 003). Animals were housed and bred according to institutional, Dutch and U.S. governmental guidelines.

### **iPSC lines**

Five iPSC lines from unrelated individuals with no diagnosed disease status were used for this study. An additional iPSC line of the same genetic background as line C2 was used, in which the NGN2 overexpression cassette was engineered into a safe harbour-locus. Details for each line, such as source material and reprogramming method are listed in the table below. iPSCs were routinely tested for mycoplasma contamination.

| **iPSC Lines** | | | | |
| --- | --- | --- | --- | --- |
| **Name in study** | **Cell line information** | **Reprogramming technique** | **Source** | **Identifier** |
| C1 | GM25256. Male skin fibroblast, 30 yo healthy control, Asian. Episomal reprogramming. | Episomal | Coriell Institute |  |
| C2 | BIONi010-C-6. Male fibroblast, 15-19y, Black/African American. Episomal reprogramming. | Episomal | Bioneer | Biosamples ID SAMEA4454009 |
| C3 | GM23973. Male skin fibroblast, 19 yo healthy control, Caucasian. Viral reprogramming | Viral | Coriell Institute, (G. Nadadhur et al., 2019) |  |
| C4 | hVS-88. Male foreskin fibroblast. Viral reprogramming. | Viral | (Holmes & Heine, 2017) |  |
| C5 | hVS-228. Female skin fibroblast, 19 yo healthy control. Viral reprogramming. | Viral | (G. Nadadhur et al., 2019) |  |
| Safe harbour NGN2 | BIONi010-C-13. Male fibroblast, 15-19y, Black/African American. Episomal reprogramming. | Episomal | Bioneer | Biosamples ID SAMEA103988285 |

## **Method Details**

### **SNP analysis and CNV calling for iPSC lines**

Prior to induction of NGN2, iPSCs were subjected to SNP-array analysis to confirm absence of large-scale genomic aberrations. DNA was prepared using QIAGEN DNeasy Blood & Tissue kit and processed by the Infinium global screening array (Illumina). CNV calling was performed using the iPsychCNV package in R (Bertalan And Hoeffding 2017, <http://biopsych.dk/iPsychCNV>). CNVs larger than 500Kb (containing >100 SNPs) were called if the region was flagged by two analysis metrics, one based on the Log R ratio and one using B allele frequency. Subsequently, called CNVs are compared against gene lists compiled based on gene ontology (GO) terms for ‘brain development’ and ‘synapse’. If genes affected by an identified CNV also occur in this gene list, the sample was excluded.

### **Generation of iPSC-derived neurons**

Induced pluripotent stem cells were maintained in Essential E8 medium (Gibco #A1517001), supplemented with 0.1% Pen/Strep (Life Technologies #11548876), on Matrigel-coated plates (VWR # BDAA256277) and passaged using Gentle Cell Dissociation Reagent (Stemcell Technologies # 07174). Upon replating, Essential E8 medium was supplemented with 5µM ROCK Inhibitor Y27632 (RI; TetuBio # T1725). Neuronal differentiation was induced as described previously (Nehme et al., 2018). To express NGN2 under control of a Tet-controlled system, iPSCs were infected in suspension (E8 medium + RI) with lentiviral vectors encoding pTet-O-Ngn2-puro (Addgene #52047) and FUΔGW-rtTa (Addgene #19780). Infected iPSCs were subsequently expanded for several passages (maximum of five) to obtain a large batch of iPSCs with the NGN2 cassette and to eliminate presence of active viral particles. To induce neurons, cells were grown in N2-supplemented medium (DMEM/F12 medium (Life Technologies # 10565018), supplemented with 200mM Glutamax (Life Technologies # 1157446), 20% Dextrose (Life Technologies # A2494001), 1% N2 supplement B (Stemcell Technologies # 07156) and 0.1% Pen/Strep) to which doxycycline hyclate (2µg/ml; Sigma # D9891) and dual SMAD inhibitors (100nM LDN193189 (Stemgent #04-0074), 10µM SB431542 (Tocris # 1614), 2µM XAV939 (Stemgent # 04-00046) were added. After 24 hours of induction, culture medium was replaced with the same components but including puromycin (Merck/Millipore # 540222) as a selection step (puromycin concentration required was determined separately for each iPSC line). This step was repeated for another 24 hours. The next day, medium was replaced by N2-supplemented culture medium with 10µM FUDR (Sigma # F0503) for another 24 hours, after which neurons were replated onto glial microdot arrays (1k per 18mm coverslip; for morphology and electrophysiology, as in (Meijer et al., 2019), 6-well plates with glia feeder layer (300k; for co-culture proteomics), or poly-ornithine (Sigma # P6407)/laminin (Sigma # L2020)-coated (300k; for neuron-only proteomics). The induction of safe-harbour iPSC-derived neurons was performed using a similar protocol, leaving out the lentiviral infection and puromycin steps. From all iPSC lines, neurons could reliably be induced, though line C5 produced a lower neuronal yield, so that insufficient numbers of neurons could be produced for electrophysiological and proteomic analyses.

The induction process was repeated several times generating several culture batches from which data were acquired. iPSC-derived neurons were maintained in neuronal maintenance medium (Neurobasal medium (Life Technologies # 11570556) including 200mM Glutamax , 20% Dextrose, Non-Essential Amino Acids (NEAA; Life Technologies # 11350912), B27 (Life Technologies # 17504044, 0.1% P/S, 0.5% Fetal bovine serum (Life Technologies # 10270106), 10ng/ml BDNF (Peprotech # 450-10), 10ng/ml CNTF (Stem Cell Technologies # 78010.1), 10ng/ml GDNF (Peprotech # 450-10), 2µg/ml Doxycycline hyclate) at 37 degrees Celsius (5.0% CO2). Medium was replaced with 50% fresh medium once (glial microdot plates) or twice (mass cultures) a week. After 39-45 days *in vitro*, at a time point where iPSC-derived neurons reliably show mature synaptic transmission (Meijer et al., 2019), samples were obtained for proteomic profiling, coverslips with autaptic neurons were fixed for morphological analysis, or patch-clamp electrophysiological recordings were performed (see subsequent sections for details).

### **Mass spectroscopy**

Cells were washed 2 times with ice cold PBS. 500 uL protease inhibitor (PI) solution in PBS (complete EDTA-free PI tablets, Roche 05056489001) was added to each well. Cells were collected by gentle scraping and spinned down for 5 minutes at 3000 rcf at 4C. Supernatant was removed and the pellet was resuspended in 20uL loading buffer (4% SDS, 100mM Tris pH 6.8, 0.04% bromophenol blue, 200mM DTT, 20% glycerol, and PI in PBS). Samples were snap frozen and stored at -80C until further processed. An SDS–PAGE LC‐MS/MS approach was used for protein identification as described previously (<https://doi-org.vu-nl.idm.oclc.org/10.1007/978-1-4939-9662-9_11>). In brief, after the sample was run into the gel, the protein-containing gel piece was excised and digested with trypsin/Lys-C mix (Promega). Peptides were analyzed by a data-independent acquisition (SWATH) method in a TripleTop 5600+ mass spectrometer (Sciex).

A spectral library was made from pooled samples of all three lines, one for each culture condition, collected at two different time points (DIV15 and DIV42). Additionally, a pooled sample glia cultured without neurons, collected at DIV15 and DIV42, was included. Spectral library samples were measured in DDA mode and analyzed using MaxQuant 1.6.3.4 (Cox & Mann, 2008). The Uniprot human reference proteome database (SwissProt + TrEMBL, version 2019-11) was used to annotate spectra. The minimum peptide length was set to 6, with at most two miss-cleavages allowed. Methionine oxidation and N-terminal acetylation were set as variable modiﬁcations with cysteine Propionamide set as ﬁxed modiﬁcation. For both peptide and protein identiﬁcation a false discovery rate of 0.01 was set.

SWATH data were searched against the spectral library (peptides and proteins identified from DDA data by MaxQuant) using Spectronaut 13.7 (Bruderer et al., 2015) with default settings. The resulting abundance values and qualitative scores for each peptide in the spectral library were exported for further downstream analysis.

**MS proteomics data analysis**

MS proteomics data analyses were performed using the R language for statistical computing. MS-DAP 0.2.6.4 (<https://github.com/ftwkoopmans/msdap>) was used for the interpretation of data quality and differential expression analysis (DEA). While importing the Spectronaut data report, fragmentgroup MS2 total peak areas without Spectronaut normalization were selected to represent peptide intensity values and both proteins from the MaxQuant contaminant database and iRT peptides were removed from the dataset.

In each statistical contrast, peptides observed in both sample groups with Spectronaut confidence score <= 0.01 in at least 2 samples (biological replicates) were selected. MS-DAP’s mode-between normalization was then applied to this data subset and finally the MSqRob statistical model was used for differential testing with “culture batch” as random variable (Goeminne, Gevaert and Clement, 2016). The significance threshold was set at 1% FDR. All data visualizations and MS-DAP parameters are included in the MS-DAP report.

To compare the protein detection between the two culture conditions (neuron-only and co-culture), we created and compared a list of proteins detected in each condition. A protein was considered “detected” if at least one peptide in one sample within that condition had a confidence score of <= 0.01. These lists were filtered for SynGO-annotated proteins (Koopmans *et al.*, 2019) to compare synaptic protein detection between the two culture conditions.

Functional enrichment analysis on the uniquely detected proteins per culture condition was performed using g:GOSt in g:Profiler (Raudvere et al., 2019) and significant hits were reported (Fig. S1C).

### **Immunocytochemistry and morphological analysis**

Neurons were fixed between DIV39-45 with 3.7% paraformaldehyde (PFA; Electron Microscopy Sciences), then washed three times with PBS pH = 7.4. Cultures were permeabilized with 0.5% Triton X-100 (Thermo Fisher #T/3751/08), followed by a 30 min incubation in PBS containing 2% normal goat serum (NGS; Thermo Fisher #11540526) and 0.1% Triton X-100. Next, neurons were stained with primary antibodies for 2h at room temperature (RT). The following antibodies were used: chicken anti-MAP2 (1:500, Abcam Ab5392) and Synaptophysin 1 (1:1000, Synaptic Systems #101004). After three washes with PBS, neurons were stained with secondary antibodies Alexa Fluor (1:1000; Invitrogen) for 1h at RT. Following three additional washes, coverslips were mounted on microscopic slides with Mowiol-DABCO. Images were acquired on a Nikon Ti-Eclipse microscope equipped with a confocal scanner model A1R+, using a 40X oil immersion objective (NA=1.3; Carl Zeiss). Z stacks were acquired with 0.5 µM intervals. Confocal settings were kept constant between cultures. Z Stacks were collapsed to maximal projections for image analysis. Images were analysed in MATLAB with SynD (Schmitz et al., 2011). Synapse detection settings were kept the same between cultures.

### **Electrophysiology**

Autaptic neurons were recorded in whole-cell voltage clamp mode using a Multiclamp 700B/AxoPatch 200B amplifier (Molecular Devices) with Digidata 1440A/1550B and Clampex 10 software (Molecular Devices). Borosilicate glass pipettes (2.5-4.5MOhm) were filled with intracellular solution (136mM KCl, 17.8mM HEPES, 1mM EGTA, 0.6mM MgCl_2_.6H_2_O, 4mM ATP-Mg, 0.3mM GTP-Na, 12mM phosphocreatine dipotassium salt, 50 units/mL phosphocreatine kinase, pH 7.3, osmolarity ~300mOsm). Recordings were made at room temperature using external solution containing 140mM NaCl, 2.4mM KCl, 2mM CaCl_2_, 4mM MgCl_2_, 10mM HEPES, 10mM glucose (pH adjusted to 7.3 with NaOH; 300mOsm). Resting membrane potential was measured in current-clamp immediately after break-in of the membrane. After this, neurons were maintained in voltage-clamp configuration at a holding potential of -70mV. Spontaneous activity was recorded first, with a sampling frequency of 20kHz (Bessel filter 5-6kHz) to allow accurate quantification of the kinetics parameters of the spontaneous events. Subsequently, a series of stimulation protocols was recorded (sampling frequency 10kHz, Bessel filter 2kHz): first the first evoked EPSC, followed by a paired-pulse (50ms inter-pulse interval) and a series of short train stimulations (5, 10, 20Hz) to induce synaptic depression. The amplitude of the 5^th^ response over the 1^st^ was used as an indicator of synaptic depression. The amplitude of the synaptic response 2 seconds after the short train was normalized to the first response of the train as an indicator of recovery after synaptic depression. Finally, 80 action potentials at 40Hz were applied to fully deplete the readily releasable vesicle pool (RRP). Recovery after pool depletion was assessed by single action potential pulses at 2 and 60 seconds post-train, respectively. Size of the RRP was estimated by back-extrapolation (Neher, 2015), and in addition the total charge transferred during the high-frequency stimulation was assessed.

Action potential stimulations were elicited by a 1ms step to +30mV. Recordings were accepted for analysis if the leak current did not exceed -300pA and the series resistance remained <15 MΩ. Subsequent selection between ‘typical’ and ‘atypical’ responses to allow adequate analysis of short-term plasticity protocols is described in supplementary figure 6. RMP and mEPSC analysis was performed for all neurons meeting aforementioned criteria, regardless of typicality of evoked responses. All offline analysis was performed using in-house developed Matlab scripts and Clampfit (Molecular Devices, v. 10.7).

### **Selection of typical versus atypical responses in electrophysiological recordings**

An algorithm was designed to quantify the relative contribution of any additional peaks. Briefly, the main peak is detected as well as any additional peaks. The ratio of the amplitude of ‘extra’ peaks over the amplitude of the main peak was then taken (total deviation ratio, TDR) for each EPSC response (schematic illustration; Fig. S6A, typical examples of algorithm output; Fig. S6B). A histogram (Fig. S6) shows the distribution of TDR values for all cells in the dataset (inset showing a zoom-in of the first bins). Simultaneously, a user visually inspected each trace and made a decision to include or exclude each recording. TDR values for user-excluded cells are substantially larger than for user-included cells (Fig. S6D). To choose a cut-off criterion, a new histogram (bins up to TDR of 0.2) split by user decision was plotted, showing that up until a TDR of maximally 0.1, more ‘user-included’ cells would be included than user-excluded cells. Thus, this cut-off criterion was applied so that all neurons giving a first evoked response with a TDR of maximum 0.1 were included for analysis of evoked synaptic transmission. Percentages of evoked response traces that were included for further analysis were comparable between neuron lines. As a comparison, the total charge transferred during the first evoked response for all recordings (i.e., without any selection) was compared to the same parameter measured in only the selected recordings. The results are highly comparable (Fig. S3F versus Fig. S6F).

### **Analysis of variation and comparison to published mouse autapse datasets**

Coefficient of variation (CoV) was calculated as standard deviation divided by the mean value, for each morphological and electrophysiological parameter of interest. For the proteomics data, the CoV was calculated for each protein and the median CoV per iPSC line was compared to published datasets. For comparison to the current dataset, values from several published mouse autapse datasets were used. For morphological parameters: mouse 1, (Lammertse et al., 2020) ; mouse 2, (Schmitz et al., 2016) ; mouse 3, (Wierda, Toonen, de Wit, Brussaard, & Verhage, 2007); mouse 4, (Emperador-Melero et al., 2018); mouse 5 (Classen, Saarloos, Meijer, Sullivan, & Verhage, 2020); for electrophysiology parameters: mouse 1, (Lammertse et al., 2020); mouse 2, (Meijer, Cijsouw, Toonen, & Verhage, 2015); mouse 3, (Meijer et al., 2018); mouse 4, (Wierda et al., 2007); mouse 5, (Emperador-Melero et al., 2018); mouse 6, (Classen et al., 2020), for proteomics: mouse 1, (He et al., 2017); mouse 2, (Vazquez-Sanchez, Gonzalez-Lozano, Walfenzao, Li, & van Weering, 2020); mouse 3, (Rosato et al., 2021); mouse 4a, (van Oostrum et al., 2020) median CoV across all DIVs; mouse 4b, (van Oostrum et al., 2020) median CoV across three conditions. To compare the variation in the current study to previously published autapse iPSC-derived neuron studies, we calculated CoVs for morphology and electrophysiology parameters from the following studies and cell lines: Study 1, (Meijer et al., 2019), C1: C14m, C2: C35m; Study 2, (Fenske et al., 2019), C1: BIHi004, C2: BIHi001; Study 3, (Rhee et al., 2019). A Kruskal-Wallis ANOVA showed that there was no significant difference between the mean CoVs of the four control lines and the safe-harbor NGN2 line measured with electrophysiology in this study (p= 0.5194).

**Analysis of explained variation**

The proportions of variance explained by culture batch were calculated by the R^2^_marginal_ (Nakagawa, Johnson, & Schielzeth, 2017). The R^2^_marginal_ quantifies the proportion of variance explained by fixed factors in the model (in our model, culture batch) in a multi-level random effects model. For the proteomics data, proportions of explained variance were calculated at the protein-level for both culture and conditions using the Bioconductor-package variancePartition in R (Hoffman & Schadt, 2016) (Fig. 3C).

**Quantification and statistical analysis**

Graphs were generated using GraphPad Prism (v. 8). Unless otherwise specified, boxplots show the median value, interquartile range, and whiskers including all values within 1.5 times IQR from the median (Tukey-style whiskers). Outliers, defined as values >3 standard deviations above or below the group mean, were excluded prior to analysis. For statistical analyses, data were standardized to meet the assumptions for linear mixed-effects models. Linear mixed-effects models were fitted to the standardized data using the *lme4* package in R (R version 3.6.3). Culture batch was included as a fixed factor in the model. The relative proportions of variance explained by the multilevel model were calculated using the MuMIn R package. The conditional intraclass correlation coefficient (conditional ICC, i.e., the ICC obtained from the model in which the batch effect is accounted for; (Nakagawa et al., 2017) was calculated using the ‘icc’ function (*performance* package in R). ICC values deviating from 0 suggest that the variation in the total data set is at least partly due to the clusters having different means (as expressed by a non-zero intercept variance). The significance of the ICC was evaluated by testing the significance of the intercept variance using a chi-square test of which the p-value is divided by two (common when testing a variance term; (Dominicus, Skrondal, Gjessing, Pedersen, & Palmgren, 2006)). For all tests, α was set to .05.

## **Power analysis**

Synapse density was selected as an example parameter for all power simulations. Synapse density data measured in this study were corrected for variation of culture batch, using a mean-centering method: each datapoint within a batch was subtracted from the mean of that batch and added with the overall mean of the dataset. This allowed simplifying the statistical models as described in textbox 2 by removing the fixed factor “Batch” from the statistical model used in the power simulations. All power curves were plotted using the ggplot2 R-package (Wickham, 2016).

*Design 1*

To select informative effect sizes for design 1, a dataset was simulated in which an experimental condition expresses a 15%, 50% and 70% difference in mean from the measured mean. Subsequently, Cohen’s *d* values were calculated by this simulated mean difference divided by the batch-corrected standard deviation measured in the present dataset. This yielded Cohen’s *d* values of 0.3299812, 1.0999372, and 1.5399121 respectively.

The ICC values observed for all synaptic parameters (Fig. 3) were plotted and three values representative of the full range of observed ICC values were selected for the power simulations: 0.01, 0.15 and 0.35. Note that the ICC_conditional_ of the measured data, corresponds to the ICC_adjusted_ in the simulated dataset. Thus, using the ICC_conditional_ of the present datasets includes correction for the effect of culture batch, which is therefore not separately modelled in the power analysis.

To simulate a case-control study, a multi-level data structure was created in which 400 data points were randomly sampled from a normal distribution with mean = 0 and sd = 1. 100 observations from the same distribution were assigned to one of two iPSC lines for each condition to ensure the variability within each condition was equal. Next, a linear mixed model for the simulated dataset was created with “condition” as a fixed variable, and “line” as a random variable, using the lme4 R-package (Bates, Mächler, Bolker, & Walker, 2015). To change the effect size between the case and control groups, the fixef() function from the SIMR package was used to assign the Cohen’s d value corresponding to the mean-difference of interest to the fixed effect “condition”. We varied the effect size (Cohen’s *d*), the variance of the random factor (such that it corresponded to the selected ICCs), the number of lines per condition and the number of observations per line. The residual variance was set at 1. For each combination of settings, 1000 power simulations were performed using the SIMR R-package (Green & MacLeod, 2016) applying an approximate F-test based on the Kenward-Roger approach with a significance threshold of alpha = 0.05. In the case of performing multiple statistical tests, this significance threshold may be adjusted to correct for this, which will impact the attainable statistical power. To assess the effect of multiple testing correction, the alpha value can be adjusted to re-run the power simulations using the R scripts provided with this paper.

*Design 2A*

Batch-corrected data of synapse density were used to calculate the coefficient of variation for each line. The mean and SD of the lines with the highest (C2) and lowest (C1) CoV were used to calculate effect sizes corresponding to 15%, 30% and 50% mean difference, which resulted in Cohen’s *d* values of 0.2900267, 0.5800533, and 0.9667555 for the high variable line and 0.4291361, 0.8582722, 1.4304536 for the low variable line.

A starting data structure was created with 400 randomly sampled data points from a normal distribution with mean = 0 and SD = 1. 200 observations were assigned to each condition. A linear model for the simulated dataset was created with “condition” as fixed effect was created using the stats R-package. We varied the effect size (Cohen’s *d*) and the number of observations per line. For each combination of settings, 1000 power simulations were performed using the SIMR R-package (Green & MacLeod, 2016) applying a t-test with a significance threshold of alpha = 0.5.

*Design 2B*

To perform power simulations on an ANOVA model, Superpower R-package was used (Lakens & Caldwell, 2021). To create the ANOVA designs, the mean and SD of the lines with the highest (C2) and lowest (C1) COV were used. Experimental means with a 15%, and 30% difference were simulated and different combinations of these means in a three-group design were entered into the ANOVA_design function. The plot_power function was used to create power curves.

*Design 3*

To simulate a multiple isogenic pairs study design, a multi-level data structure was created in which 400 data points were randomly sampled from a normal distribution with mean = 0 and sd = 1. 100 observations were assigned to one of two iPSC lines for each condition and both lines were included in each condition (case and control). Next, a linear mixed model for the simulated dataset was created with “condition” as a fixed variable, “line” as a random intercept variable, and “condition” as a random slope variable using the lme4 R-package (Bates et al., 2015). The residual variance was set at 1 and the variance of the random intercept factor “line” was set such that it corresponded to an ICC of 0.35. We simulated two effect sizes (Cohen’s *d*) of 0.3299812 and 1.0999372, corresponding to a mean difference in our dataset of 15% and 50% respectively. Four different values for the variance of the random slope factor “condition’ were simulated: 0.001, 0.05, 0.15, and 0.5. The number of lines and the number of observations per line per condition were varied as for Design 1. For each combination of settings, 1000 power simulations were performed using the SIMR R-package (Green & MacLeod, 2016) applying an approximate F-test based on the Kenward-Roger approach with a significance threshold of alpha = 0.5.

**Supplementary references**

Bates, D., Mächler, M., Bolker, B. M., & Walker, S. C. (2015). Fitting linear mixed-effects models using lme4. *Journal of Statistical Software*, *67*(1). https://doi.org/10.18637/jss.v067.i01

Bruderer, R., Bernhardt, O. M., Gandhi, T., Miladinović, S. M., Cheng, L. Y., Messner, S., … Reiter, L. (2015). Extending the limits of quantitative proteome profiling with data-independent acquisition and application to acetaminophen-treated three-dimensional liver microtissues. *Molecular and Cellular Proteomics*, *14*(5), 1400–1410. https://doi.org/10.1074/mcp.M114.044305

Classen, J., Saarloos, I., Meijer, M., Sullivan, P. F., & Verhage, M. (2020). A Munc18-1 mutant mimicking phosphorylation by Down Syndrome-related kinase Dyrk1a supports normal synaptic transmission and promotes recovery after intense activity. *Scientific Reports*, *10*(1), 3181. https://doi.org/10.1038/s41598-020-59757-y

Cox, J., & Mann, M. (2008). MaxQuant enables high peptide identification rates, individualized p.p.b.-range mass accuracies and proteome-wide protein quantification. *Nature Biotechnology*, *26*(12), 1367–1372. https://doi.org/10.1038/nbt.1511

Dominicus, A., Skrondal, A., Gjessing, H. K., Pedersen, N. L., & Palmgren, J. (2006). Likelihood ratio tests in behavioral genetics: Problems and solutions. *Behavior Genetics*, *36*(2), 331–340. https://doi.org/10.1007/s10519-005-9034-7

Emperador-Melero, J., Huson, V., van Weering, J., Bollmann, C., Fischer von Mollard, G., Toonen, R. F., & Verhage, M. (2018). Vti1a/b regulate synaptic vesicle and dense core vesicle secretion via protein sorting at the Golgi. *Nature Communications*, *9*(1), 1–17. https://doi.org/10.1038/s41467-018-05699-z

Fenske, P., Grauel, M. K., Brockmann, M. M., Dorrn, A. L., Trimbuch, T., & Rosenmund, C. (2019). Autaptic cultures of human induced neurons as a versatile platform for studying synaptic function and neuronal morphology. *Scientific Reports*, *9*(1), 1–12. https://doi.org/10.1038/s41598-019-41259-1

G. Nadadhur, A., Alsaqati, M., Gasparotto, L., Cornelissen-Steijger, P., van Hugte, E., Dooves, S., … Heine, V. M. (2019). Neuron-Glia Interactions Increase Neuronal Phenotypes in Tuberous Sclerosis Complex Patient iPSC-Derived Models. *Stem Cell Reports*, *12*(1), 42–56. https://doi.org/10.1016/j.stemcr.2018.11.019

Green, P., & MacLeod, C. J. (2016). SIMR: An R package for power analysis of generalized linear mixed models by simulation. *Methods in Ecology and Evolution*, *7*(4), 493–498. https://doi.org/10.1111/2041-210X.12504

He, E., Wierda, K., Van Westen, R., Broeke, J. H., Toonen, R. F., Cornelisse, L. N., & Verhage, M. (2017). Munc13-1 and Munc18-1 together prevent NSF-dependent de-priming of synaptic vesicles. *Nature Communications*, *8*(May), 1–10. https://doi.org/10.1038/ncomms15915

Hoffman, G. E., & Schadt, E. E. (2016). variancePartition: Interpreting drivers of variation in complex gene expression studies. *BMC Bioinformatics*, *17*(1), 17–22. https://doi.org/10.1186/s12859-016-1323-z

Holmes, D. B., & Heine, V. M. (2017). Simplified 3D protocol capable of generating early cortical neuroepithelium. *Biology Open*, *6*(3), 402–406. https://doi.org/10.1242/bio.021725

Lakens, D., & Caldwell, A. R. (2021). Simulation-Based Power Analysis for Factorial Analysis of Variance Designs. *Advances in Methods and Practices in Psychological Science*, *4*(1), 1–11. https://doi.org/10.1177/2515245920951503

Lammertse, H. C. A., Van Berkel, A. A., Iacomino, M., Toonen, R. F., Striano, P., Gambardella, A., … Zara, F. (2020). Homozygous STXBP1 variant causes encephalopathy and gain-of-function in synaptic transmission. *Brain*, *143*(2), 441–451. https://doi.org/10.1093/brain/awz391

Meijer, M., Cijsouw, T., Toonen, R. F., & Verhage, M. (2015). Synaptic effects of Munc18-1 alternative splicing in excitatory hippocampal neurons. *PLoS ONE*, *10*(9), 1–14. https://doi.org/10.1371/journal.pone.0138950

Meijer, M., Dörr, B., Lammertse, H. C., Blithikioti, C., Weering, J. R., Toonen, R. F., … Verhage, M. (2018). Tyrosine phosphorylation of Munc18‐1 inhibits synaptic transmission by preventing SNARE  assembly . *The EMBO Journal*, *37*(2), 300–320. https://doi.org/10.15252/embj.201796484

Meijer, M., Rehbach, K., Brunner, J. W., Classen, J. A., Lammertse, H. C. A., van Linge, L. A., … Verhage, M. (2019). A Single-Cell Model for Synaptic Transmission and Plasticity in Human iPSC-Derived Neurons. *Cell Reports*, *27*(7), 2199-2211.e6. https://doi.org/10.1016/j.celrep.2019.04.058

Nakagawa, S., Johnson, P. C. D., & Schielzeth, H. (2017). The coefficient of determination R2 and intra-class correlation coefficient from generalized linear mixed-effects models revisited and expanded. *Journal of the Royal Society Interface*, *14*(134). https://doi.org/10.1098/rsif.2017.0213

Nehme, R., Zuccaro, E., Ghosh, S. D., Li, C., Sherwood, J. L., Pietilainen, O., … Eggan, K. (2018). Combining NGN2 Programming with Developmental Patterning Generates Human Excitatory Neurons with NMDAR-Mediated Synaptic Transmission. *Cell Reports*, *23*(8), 2509–2523. https://doi.org/10.1016/J.CELREP.2018.04.066

Raudvere, U., Kolberg, L., Kuzmin, I., Arak, T., Adler, P., Peterson, H., & Vilo, J. (2019). G:Profiler: A web server for functional enrichment analysis and conversions of gene lists (2019 update). *Nucleic Acids Research*, *47*(W1), W191–W198. https://doi.org/10.1093/nar/gkz369

Rhee, H. J., Shaib, A. H., Rehbach, K., Lee, C. K., Seif, P., Thomas, C., … Rhee, J. S. (2019). An Autaptic Culture System for Standardized Analyses of iPSC-Derived Human Neurons. *Cell Reports*, *27*(7), 2212-2228.e7. https://doi.org/10.1016/j.celrep.2019.04.059

Rosato, M., Stringer, S., Gebuis, T., Paliukhovich, I., Li, K. W., Posthuma, D., … van Kesteren, R. E. (2021). Combined cellomics and proteomics analysis reveals shared neuronal morphology and molecular pathway phenotypes for multiple schizophrenia risk genes. *Molecular Psychiatry*, *26*(3), 784–799. https://doi.org/10.1038/s41380-019-0436-y

Schmitz, S. K., Hjorth, J. J. J., Joemai, R. M. S., Wijntjes, R., Eijgenraam, S., de Bruijn, P., … Veldkamp, W. (2011). Automated analysis of neuronal morphology, synapse number and synaptic recruitment. *Journal of Neuroscience Methods*, *195*(2), 185–193. https://doi.org/10.1016/j.jneumeth.2010.12.011

Schmitz, S. K., King, C., Kortleven, C., Huson, V., Kroon, T., Kevenaar, J. T., … Toonen, R. F. (2016). Presynaptic inhibition upon CB 1 or mG lu2/3 receptor activation requires ERK / MAPK phosphorylation of Munc18‐1 . *The EMBO Journal*, *35*(11), 1236–1250. https://doi.org/10.15252/embj.201592244

van Oostrum, M., Campbell, B., Seng, C., Müller, M., tom Dieck, S., Hammer, J., … Wollscheid, B. (2020). Surfaceome dynamics reveal proteostasis-independent reorganization of neuronal surface proteins during development and synaptic plasticity. *Nature Communications*, *11*(1), 1–16. https://doi.org/10.1038/s41467-020-18494-6

Vazquez-Sanchez, S., Gonzalez-Lozano, M. A., Walfenzao, A., Li, K. W., & van Weering, J. R. T. (2020). The endosomal protein sorting nexin 4 is a synaptic protein. *Scientific Reports*, *10*(1), 1–12. https://doi.org/10.1038/s41598-020-74694-6

Wickham, H. (2016). *ggplot2: Elegant Graphics for Data Analysis*. Springer-Verlag New York. Retrieved from https://ggplot2.tidyverse.org

Wierda, K. D. B., Toonen, R. F. G., de Wit, H., Brussaard, A. B., & Verhage, M. (2007). Interdependence of PKC-Dependent and PKC-Independent Pathways for Presynaptic Plasticity. *Neuron*, *54*(2), 275–290. https://doi.org/10.1016/j.neuron.2007.04.001
