# Supplementary material for: Power and optimal study design in iPSC-based brain disease modelling
Source: Mol Psychiatry. 2022 Nov 16;28(4):1545–56. doi: 10.1038/s41380-022-01866-3 (PMC10208961; doi:10.1038/s41380-022-01866-3)
Supplement: Supplementary file 3 — Supplemental table 2: R2 values (proportion of variance explained), calculated as described in Methods section. [file 41380_2022_1866_MOESM3_ESM.docx]

**Table 2: R^2^ values (proportion of variance explained), calculated as described in Methods section.**

| **Parameter** | **R^2^ marginal** | **R^2^ conditional** | **R^2^ marginal + conditional** | **R^2^ residual** |
| --- | --- | --- | --- | --- |
| Dendrite length | 0.103 | 0.117 | 0.117 | 0.883 |
| Synapse density | 0.0389 | 0.274 | 0.274 | 0.726 |
| Synapse intensity | 0.127 | 0.327 | 0.327 | 0.674 |
| Soma area | 0.041 | 0.048 | 0.048 | 0.952 |
| Resting membrane potential | 0.035 | 0.020 | 0.0548 | 0.945 |
| mEPSC frequency | 0.012 | 0.012 | 0.013 | 0.988 |
| mEPSC amplitude | 0.037 | 0.037 | 0.038 | 0.962 |
| EPSC amplitude | 0.066 | 0.232 | 0.232 | 0.769 |
| EPSC charge | 0.002 | 0.328 | 0.328 | 0.672 |
| Paired-pulse ratio | 0.054 | 0.159 | 0.159 | 0.841 |
| 5Hz Synaptic depression | 0.051 | 0.197 | 0.197 | 0.803 |
| 5Hz Recovery | 0.023 | 0.101 | 0.101 | 0.899 |
| 10Hz Synaptic depression | 0.021 | 0.102 | 0.102 | 0.898 |
| 10Hz Recovery | 0.061 | 0.065 | 0.065 | 0.935 |
| 20Hz Synaptic depression | 0.039 | 0.120 | 0.121 | 0.880 |
| 20Hz Recovery | 0.056 | 0.076 | 0.076 | 0.924 |
| 40Hz Recovery (2 sec.) | 0.010 | 0.351 | 0.351 | 0.649 |
| 40Hz Recovery (60 sec.) | 0.017 | 0.0166 | 0.017 | 0.9833 |
| 40Hz Total charge | 0.051 | 0.165 | 0.166 | 0.835 |
| RRP estimate | 0.057 | 0.062 | 0.062 | 0.936 |
