# Supplementary material for: Power and optimal study design in iPSC-based brain disease modelling
Source: Mol Psychiatry. 2022 Nov 16;28(4):1545–56. doi: 10.1038/s41380-022-01866-3 (PMC10208961; doi:10.1038/s41380-022-01866-3)
Supplement: Supplementary file 4 — Supplemental Table 1: Coefficient of variation (CoV) values [file 41380_2022_1866_MOESM4_ESM.docx]

**Table 1**: **Coefficient of variation (CoV) values**

| **Parameter** | **Study, Experimental group** | **Mean** | **SD** | **CoV** | **Sample size (n)** |
| --- | --- | --- | --- | --- | --- |
| Dendrite length | Present study – C1 | 1311 | 478.9 | 0.365 | 54 |
|  | Present study – C2 | 1224 | 456.6 | 0.373 | 56 |
|  | Present study – C3 | 1258 | 557.6 | 0.443 | 59 |
|  | Present study – C4 | 1228 | 682.7 | 0.556 | 59 |
|  | Present study – C5 | 1107 | 728.2 | 0.658 | 54 |
|  | Mouse 1; Lammertse, v. Berkel (2019) | 1247.2561 | 533.1779 | 0.435 | 30 |
|  | Mouse 2; Schmitz (2016) | 1226 | 435.63 | 0.355 | 51 |
|  | Mouse 3; Wierda (2007) | 2126.8 | 974.26 | 0.458 | 21 |
|  | Mouse 4; Emperador-Melero (2018) | 2128.2 | 653.28 | 0.307 | 18 |
|  | Mouse 5; Classen (2020) | 1720 | 802.3 | 0.446 | 45 |
|  | Meijer et al. (2019) – C14m | 2998 | 6044 | 2.016 | 16 |
|  | Fenske et al. (2019) – BIHi001 | 377 | 352.139177 | 0.934 | 18 |
|  | Fenske et al. (2019) – BIHi004 | 253 | 164.1645516 | 0.649 | 22 |
| Synapse density | Present study – C1 | 0.1146 | 0.0416 | 0.363 | 56 |
|  | Present study – C2 | 0.0792 | 0.0358 | 0.453 | 57 |
|  | Present study – C3 | 0.0789 | 0.0327 | 0.415 | 59 |
|  | Present study – C4 | 0.0838 | 0.0348 | 0.415 | 60 |
|  | Present study – C5 | 0.0585 | 0.0263 | 0.450 | 53 |
|  | Mouse 1; Lammertse, v. Berkel (2019) | 0.4019 | 0.10357 | 0.258 | 30 |
|  | Mouse 2; Schmitz (2016) | 0.179 | 0.0571 | 0.319 | 51 |
|  | Mouse 3; Wierda (2007) |  |  | 0.381 | 21 |
|  | Mouse 4; Emperador-Melero (2018) | 0.24 | 0.0755 | 0.315 | 57 |
|  | Mouse 5; Classen (2020) | 0.2 | 0.0624 | 0.312 | 39 |
|  | Fenske et al. (2019) – BIHi001 | 0.26 | 0.140712473 | 0.541 | 22 |
|  | Fenske et al. (2019) – BIHi004 | 0.42 | 0.130766968 | 0.311 | 19 |
| mEPSC Frequency | Present study – C1 | 1.223 | 1.834 | 1.499 | 16 |
|  | Present study – C2 | 1.185 | 1.261 | 1.064 | 16 |
|  | Present study – C3 | 1.697 | 2.69 | 1.585 | 22 |
|  | Present study – C4 | 1.325 | 2.126 | 1.605 | 15 |
|  | Mouse 1; Lammertse, v. Berkel (2019) | 5.877 | 10.83 | 1.843 | 27 |
|  | Mouse 2; Meijer (2015) | 5.6 | 8.7492 | 1.562 | 46 |
|  | Mouse 3; Meijer (2017) | 20.18 | 20.062 | 0.994 | 15 |
|  | Mouse 4; Wierda (2007) | 29.3 | 23.572 | 0.805 | 14 |
|  | Mouse 5; Emperador-Melero (2018) | 15.45 | 16.292 | 1.054 | 18 |
|  | Mouse 6; Classen (2020) | 10.52 | 10.523 | 1.000 | 31 |
|  | Present study – BioniC13 | 0.9375 | 1.169 | 1.247 | 9 |
|  | Meijer et al. (2019) – c14m | 1.6 | 2.936784 | 1.835 | 63 |
|  | Meijer et al. (2019) – c35m | 0.34 | 2.0959008 | 6.164 | 38 |
|  | Rhee et al. (2019) | 3.09 | 2.563435195 | 0.830 | 12 |
| mEPSC Ampltiude | Present study – C1 | 64.18 | 83.19 | 1.296 | 15 |
|  | Present study – C2 | 39.59 | 11.31 | 0.286 | 16 |
|  | Present study – C3 | 62.87 | 103 | 1.638 | 22 |
|  | Present study – C4 | 60.91 | 50.09 | 0.822 | 15 |
|  | Mouse 1; Lammertse, v. Berkel (2019) | 21.46 | 4.65 | 0.217 | 26 |
|  | Mouse 2; Meijer (2015) | 18.11 | 5.9685 | 0.330 | 46 |
|  | Mouse 3; Meijer (2017) | 26 | 6.5841 | 0.253 | 15 |
|  | Mouse 4; Wierda (2007) | 29.1 | 10.851 | 0.373 | 14 |
|  | Mouse 6; Classen (2020) | 16 | 4.0729 | 0.255 | 32 |
|  | Present study – BioniC13 | 36.17 | 10.52 | 0.291 | 8 |
|  | Meijer et al. (2019) – c14m | 28.3 | 12.079735 | 0.427 | 57 |
|  | Meijer et al. (2019) – c35m | 21.3 | 8.6162637 | 0.405 | 29 |
|  | Fenske et al. (2019) – BIHi001 | 39.7 | 18.897619 | 0.476 | 62 |
|  | Fenske et al. (2019) – BIHi004 | 30.4 | 22.224311 | 0.731 | 63 |
|  | Rhee et al. (2019) | 34.28 | 12.47076581 | 0.364 | 12 |
| EPSC Peak | Present study – C1 | 3.494 | 2.651 | 0.759 | 14 |
| amplitude | Present study – C2 | 6.092 | 3.3 | 0.541 | 17 |
|  | Present study – C3 | 4.848 | 3.113 | 0.642 | 23 |
|  | Present study – C4 | 7.415 | 3.894 | 0.525 | 20 |
|  | Mouse 1; Lammertse, v. Berkel (2019) | 4.663 | 3.684 | 0.790 | 32 |
|  | Mouse 2; Meijer (2015) | 3.21 | 2.0795 | 0.648 | 45 |
|  | Mouse 3; Meijer (2017) | 7.96 | 5.1486 | 0.647 | 30 |
|  | Mouse 4; Wierda (2007) | 1.422 | 0.79599 | 0.559 | 11 |
|  | Mouse 6; Classen (2020) | 4.04 | 3.1426 | 0.778 | 22 |
|  | Present study – BioniC13 | 6.313 | 3.583 | 0.568 | 38 |
|  | Meijer et al. (2019) – c14m | 7.65 | 4.8648947 | 0.636 | 32 |
|  | Meijer et al. (2019) – c35m | 4.88 | 3.349791 | 0.686 | 11 |
|  | Fenske et al. (2019) – BIHi001 | 1.1 | 1.6733201 | 1.521200048 | 70 |
|  | Fenske et al. (2019) – BIHi004 | 1 | 0.83666 | 0.836660027 | 70 |
|  | Rhee et al. (2019) | 1.53 | 1.620185175 | 1.059 | 42 |
| EPSC Charge | Present study – C1 | 45.65 | 30.44 | 0.667 | 14 |
|  | Present study – C2 | 56.4 | 28.29 | 0.502 | 16 |
|  | Present study – C3 | 53.27 | 31.53 | 0.592 | 22 |
|  | Present study – C4 | 126.3 | 83.45 | 0.661 | 20 |
|  | Mouse 1; Lammertse, v. Berkel (2019) | 59.38 | 50.65 | 0.853 | 32 |
|  | Mouse 3; Meijer (2017) | 70.9 | 67.55 | 0.953 | 12 |
|  | Mouse 5; Emperador-Melero (2018) | 145.03 | 165.22 | 1.139 | 15 |
|  | Present study – BioniC13 | 53.56 | 32.36 | 0.604 | 38 |
| Paired-pulse ratio | Present study – C1 | 0.9135 | 0.4612 | 0.505 | 14 |
|  | Present study – C2 | 0.7184 | 0.2871 | 0.399 | 20 |
|  | Present study – C3 | 0.6809 | 0.2863 | 0.425 | 20 |
|  | Present study – C4 | 0.5979 | 0.2677 | 0.448 | 21 |
|  | Mouse 1; Lammertse, v. Berkel (2019) | 0.992 | 0.5002 | 0.504 | 34 |
|  | Mouse 2; Meijer (2015) | 0.89 | 0.2683 | 0.301 | 45 |
|  | Mouse 6; Classen (2020) | 1.281 | 0.5077 | 0.396 | 22 |
|  | Present study – BioniC13 | 0.8973 | 0.3093 | 0.345 | 34 |
|  | Rhee et al. (2019) | 0.65 | 0.274954542 | 0.423 | 21 |
| RRP Estimate | Present study – C1 | 111.5 | 52.77 | 0.473 | 11 |
|  | Present study – C2 | 163.5 | 117.4 | 0.718 | 16 |
|  | Present study – C3 | 95.87 | 69.19 | 0.722 | 18 |
|  | Present study – C4 | 119.2 | 108.3 | 0.909 | 15 |
|  | Mouse 1; Lammertse, v. Berkel (2019) | 569.4 | 495 | 0.869 | 23 |
|  | Mouse 2; Meijer (2015) | 377.8 | 311.78 | 0.825 | 35 |
|  | Mouse 6; Classen (2020) | 759.2 | 521.5 | 0.687 | 17 |
|  | Present study – BioniC13 | 287 | 268.4 | 0.935 | 26 |
| Proteomics: Neuron-only | Present study – C1 |  |  | 0.113 |  |
|  | Present study – C2 |  |  | 0.087 |  |
|  | Present study – C3 |  |  | 0.068 |  |
|  | Mouse 1; He (2017) |  |  |  |  |
|  | Mouse 2; Vazquez-Sanchez (2020) |  |  | 0.14 |  |
|  | Mouse 3; Rosato (2019) |  |  | 0.107 |  |
|  | Mouse 4a; v. Oostrum (2020), across all DIVs |  |  | 0.151 |  |
|  | Mouse 4b; v. Oostrum (2020), across all conditions |  |  | 0.110 |  |
| Proteomics: Neuron-glia co-culture | Present study – C1 |  |  | 0.110 |  |
|  | Present study – C2 |  |  | 0.095 |  |
|  | Present study – C3 |  |  | 0.109 |  |
|  | Total CoV (Present study) |  |  | 0.120 |  |
