# Supplementary material for: Power and optimal study design in iPSC-based brain disease modelling
Source: Mol Psychiatry. 2022 Nov 16;28(4):1545–56. doi: 10.1038/s41380-022-01866-3 (PMC10208961; doi:10.1038/s41380-022-01866-3)
Supplement: Supplementary file 5 — Supplemental textbox 1. Statistical terms [file 41380_2022_1866_MOESM5_ESM.pdf]

Box 1: Statistical terms

Type I error. In a null-hypothesis test, a Type I error (false positive) occurs if in reality an effect is absent, but the null-hypothesis is rejected. The probability to commit a Type I error is denoted by α, which is often set to .05. However, if data are clustered, and this clustering is not accounted for statistically, then the true Type I error rate can be (much) higher than the chosen α.

Type II error. In a null-hypothesis test, a Type II error (false negative) occurs if in reality an effect is present, but the null-hypothesis is not rejected. The probability to commit a Type II error is denoted by β. Low powered studies have higher Type II error rates, i.e., greater probability that true effects will go undetected. Under specific circumstances, not statistically accounting for the clustering in the data can result in an increased Type II error rate, i.e., a loss of power.

Statistical power. In a null-hypothesis test, the power is the probability to correctly reject the null hypothesis, i.e., to detect a true effect. Power is denoted 1-β, where β denotes the probability of a Type II error. Power depends on multiple factors such as the number of observations, effect size, and study design. Studies with low statistical power readily fail to detect true effects, i.e., have higher Type II error rate, or give biased estimates of true effects, thus hampering scientific progress.

Standardized effect size. A standardized, scale-free measure of the magnitude of an effect, that is comparable across studies, even when different instruments (i.e., measurement scales) are used. Various effect sizes exist. When comparing two group means (e.g., properties of iPSC neurons from cases versus controls), Cohen's *d* is the most appropriate. Cohen's *d* is calculated as the difference between the two group means divided by their pooled standard deviation.

Variance. Measure of the variability in a data set. When a study includes multiple iPSC lines from each of which multiple neurons are cultured, we can distinguish variation observed *within* a line and variation observed *between* lines. Variation within a line can e.g. be due to random measurement variation, biological variation between neurons, systematic differences between culture batches, or, in case of multiple isogenic pairs, due to the possibly differential effect of the introduction/repair of a genetic variant. Variation *between* lines is due to systematic mean differences between lines caused by e.g. the absence/presence of a genetic variant or differences in genetic background. Correcting for sources of variation such as culture batch effects, will decrease the overall variation in the data and will increase the power to detect effects of interest (i.e., differences between lines or conditions).

Clustered data. Also known as nested, multilevel, or hierarchical data. In iPSC research, multiple neurons are cultured from one iPSC line. In study designs featuring multiple lines, this results in a clustered data set, where the clusters are formed by neurons from the same line. Importantly, observations from the same cluster tend to correlate, i.e., neurons from the same line tend to be more similar to each other than to neurons from different lines.

Dependent observations. Correlated observations from the same cluster cannot be treated as independent observations in statistical analyses. The clustering or dependency in the data is introduced by the way the data are collected (e.g., including multiple neurons per line), and needs to be accommodated statistically to avoid inflated Type I error rates. Statistical tests like t-test, AN(C)OVA, and linear regression assume observations to be independent, and are thus unsuited for the analysis of clustered data.

Intracluster correlation (ICC). Also known as intraclass correlation. In clustered data, the ICC is a measure of the relative similarity of observations from the same cluster, e.g., neurons from the same iPSC line. The higher the similarity between observations from the same cluster, the stronger the dependency in the data, and the greater the need to accommodate that dependency statistically. Theoretically, ICC values range from 0 (no dependency) to 1 (full dependency). When the number of neurons per line is high, even low levels of dependency (i.e., low ICC values) require statistical accommodation (see Textbox Fig. 1).

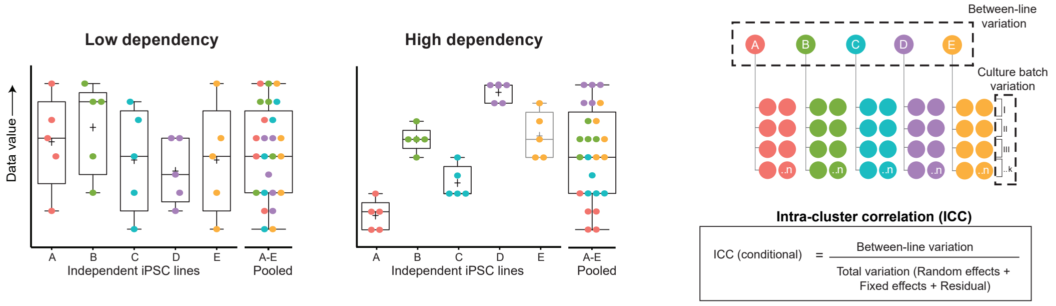

Textbox Figure 1. Dependency

Linear regression model. A linear regression model allows one to estimate the linear relationship between an outcome (dependent) variable y and one or more predictor (independent) variables. Research questions pertaining to the equality of two group means can be formulated as a linear regression model by including group membership (or condition, i.e., controls versus cases, coded 0 and 1, respectively) as predictor, i.e., $y_i = b_o + b_1 * G_i + \varepsilon_i$. Here, y_i denotes the outcome variable for neuron i, G_i the 0/1 coded group membership of neuron i, and ε_i the neuron-specific residual. Parameters b_0 and b_1 denote the intercept and slope, respectively. In the context of a 0/1-coded grouping variable G, the intercept b_0 denotes the mean of the group coded as 0, while the mean of the group coded as 1 is estimated as $b_0 + b_1$. The slope b_1 thus denotes the mean difference between the two groups. If statistical testing shows that b_1 is significantly different from 0, then this implies that controls and cases have different means. In a standard linear regression model, both b_0 and b_1 are assumed fixed, i.e., while values y_i, G_i and ε_i vary over neurons i, the parameters of b_0 and b_1 are the same for all neurons.

Fixed parameters. In the context of linear (mixed) models, fixed parameters denote parameters whose values do not vary across clusters.

Random parameters. In the context of linear mixed models, random parameters denote parameters whose values vary across clusters. Only clustered data (i.e., Designs 1 and 3) allow estimation of random parameters (see Box 2).

Linear mixed model. Also known as multi-level model. An extension of the standard linear regression model that accommodates clustered data and allows the estimation of both fixed and random parameters. In the context of clustered data, the mean of the outcome variable may differ between the K clusters (Design 1; intercept variance), and the effect of the experimental manipulation may vary across the K clusters (Design 3; slope variance). Linear mixed models accommodate such variation across clusters by allowing b_0 and/or b_1 to be random parameters, i.e., their values may vary across clusters. This involves an extension of the standard linear model: $y_{ik} = b_{ok} + b_{1k} * G_{ik} + \varepsilon_{ik}$. In contrast to linear regression model, all parameters now have a cluster-specific subscript k. Specifically, y_{ik} denotes the outcome variable for neuron i from cluster k, G_{ik} the 0/1 coded group membership of neuron i from cluster k, and ε_{ik} the neuron-and-cluster specific residual. Parameters b_{0k} and b_{1k} denote the cluster-specific intercept and cluster-specific slope, respectively.

Specifically, the linear mixed model accommodates the variation in means (i.e., intercept) and/or the variation in experimental effects (i.e., slope) across the K clusters as follows. Rather than estimating K cluster-specific means, the linear mixed model estimates the overall mean calculated across the K clusters, and a variance of this mean. If this variance term is significantly different from zero, one concludes that the K clusters indeed vary in their means. If this variance term is not significantly different from zero, then one concludes that the cluster means are equal.

Similarly, for the experimental effect, the linear mixed model estimates the mean slope (i.e., the average effect of the experimental manipulation as calculated across all K clusters) and the variance of the slope, indicative of the variation of the effect of the experimental manipulation across the K clusters. If the variance of the slope is significantly different from zero, one concludes that the effect of the experimental manipulation varies significantly across the K clusters (this is actually interpretable as a cluster-by-experimental manipulation interaction). If this variance term is not significantly different from zero, then one concludes that the experimental manipulation had the same effect in all K clusters.
