# Supplementary material for: Power and optimal study design in iPSC-based brain disease modelling
Source: Mol Psychiatry. 2022 Nov 16;28(4):1545–56. doi: 10.1038/s41380-022-01866-3 (PMC10208961; doi:10.1038/s41380-022-01866-3)
Supplement: Supplementary file 6 — Supplemental textbox 2. Data structures and appropriate statistical model [file 41380_2022_1866_MOESM6_ESM.pdf]

Box 2: Data structures and appropriate statistical model

In all iPSC study designs, multiple neurons are cultured from the same line, often in multiple culture batches. The inclusion of one isogenic line or multiple genetically heterogeneous lines does, however, result in design-specific data structures, as illustrated in Fig. TB2. These different data structures call for different statistical approaches. There are several excellent software packages that facilitate statistical analysis of such data, for example the *limma* and *lme4* R packages. For practical guidance on the use of these packages, see e.g. (Hoffman and Roussos, 2021) for analysis of gene expression data using repeated measures designs, or (Yu *et al.*, 2021) for the application of the *lme4* package to generate linear mixed models for cell biological experiments.

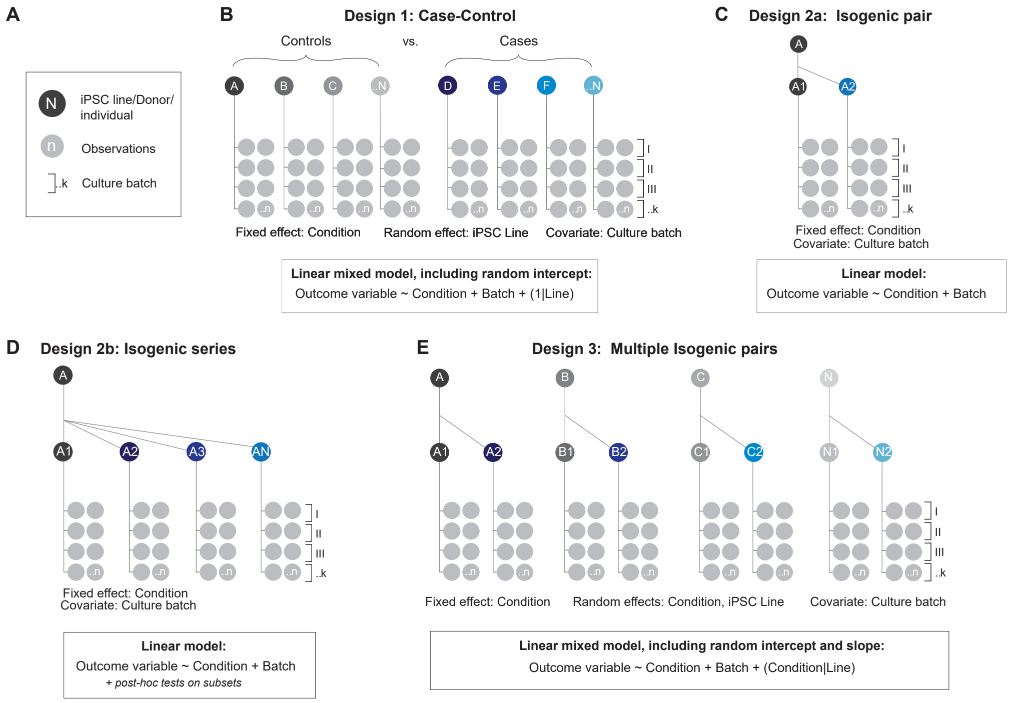

Textbox Figure 1. Data structures per design

Designs including multiple iPSC-lines

Designs 1 and 3 include multiple, genetically heterogeneous iPSC-lines. As the multiple neurons from the same line are often more similar to each other than to neurons from other lines, this data collection strategy results in clustered data, i.e., dependent observations. This clustering or dependency needs to be accounted for statistically to avoid inflated Type I error rates (Aarts *et al.*, 2014, 2015; Yu *et al.*, 2021). Linear mixed models can accommodate the clustering.

Design 1 features k control and patient lines, and from each of these lines multiple neurons are cultured (i.e., clustered data). Here, the outcome variable does not necessarily have the same mean in all iPSC-lines from the same condition (e.g., controls). In a linear mixed model, this variation in means is accommodated by allowing the intercept b_0 to vary, i.e., be random, over clusters (Fig. TB2B). Rather than estimating k cluster-specific means, a linear mixed model will estimate 1 variance term for the intercept to accommodate the variation in means across the k clusters.

Design 3, features *k* isogenic lines, from which multiple neurons are cultured before and after an experimental manipulation, e.g., introduction/repair of a genetic variant (i.e., clustered data, with an experimental manipulation within each of the *k* clusters; Fig. TB1). Like in Design 1, the outcome variable does not necessarily have the same mean in all IPSC lines from the same condition (e.g., the condition excluding the pathogenic mutation), and like in Design 1, such variation within conditions is accounted for by allowing the intercept to be a random parameter (Fig. TB2E). However, Design 3 differs from Design 1 in that the effect of the experimental manipulation on the outcome variable can be observed within each line. The effect of the experimental manipulation on the outcome variable is not necessarily the same in all *k* lines, but may vary, e.g., as a function of the genetic background within which the manipulation takes place. In a linear mixed model, this variation in the strength of the effect of the manipulation on the outcome variable is accommodated by allowing the slope b_1 to vary, i.e., be random, over clusters (Fig. TB2E). Rather than estimating *k* cluster-specific slopes, a linear mixed model will estimate 1 variance term for the slope to accommodate the variation in effect of the manipulation across the *k* clusters (see Fig. S6 for a visual illustration of intercept and slope variation in Design 3).

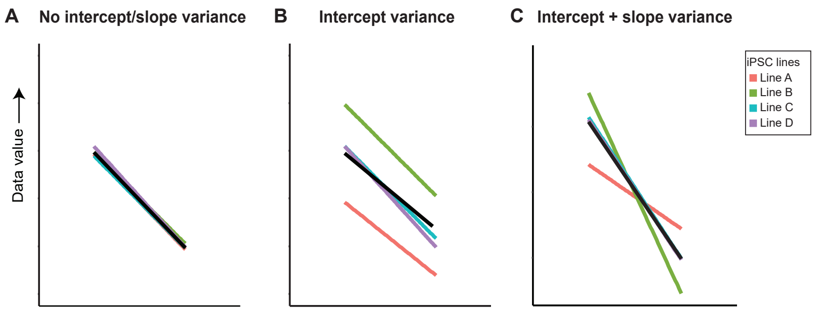

Textbox Figure 2. Random slope variance

Designs including a single isogenic line

In Designs 2A and 2B, data are collected from a single isogenic line. The culturing of multiple neurons from the same line does in this case not result in a clustered data structure because all observations belong to the very same cluster. All observations can therefore be considered independent and standard statistical techniques like t-test, AN(C)OVA, and linear regression (Fig. TB2C&D) can be applied.

Covariates

Variables that affect the outcome variable but are themselves not of direct scientific interest, need to be included as covariate in the statistical model because they can mask or bias the effect of interest. For instance, in iPSC studies, neurons are often cultured in multiple distinguishable culture batches. The multi-step process of derivation and differentiation of neurons can introduce (non)random technical variation between culture batches that affects the outcome variable and masks or bias the biological effects of interest (Schwartzentruber *et al.*, 2018; Volpato and Webber, 2020). Culture batch therefore needs to be accounted for statistically, e.g., by explicitly including it as a covariate in all statistical analyses, irrespective of the exact study design. Generally, the effect of covariates is modelled through fixed parameters. However, if clustered data are collected and one wants to allow the possibility that the effect of the covariate varies across clusters, the covariates can be included as random in the statistical model.
